# On a boundary-localized Higgs boson in 5D theories

**DOI:** 10.1140/epjc/s10052-015-3756-3

**Published:** 2015-11-05

**Authors:** Roberto Barceló, Subhadip Mitra, Grégory Moreau

**Affiliations:** Laboratoire de Physique Théorique, Université Paris-sud 11 et CNRS-UMR 8627, 91405 Orsay Cedex, France

## Abstract

In the context of a simple five-dimensional (5D) model with bulk matter coupled to a brane-localized Higgs boson, we point out a non-commutativity in the 4D calculation of the mass spectrum for excited fermion towers: the obtained expression depends on the choice in ordering the limits, $N \rightarrow \infty$ (infinite Kaluza–Klein tower) and $\epsilon \rightarrow 0$ ($\epsilon$ being the parameter introduced for regularizing the Higgs Dirac peak). This introduces the question of which one is the correct order; we then show that the two possible orders of regularization (called I and II) are experimentally equivalent, as both can typically reproduce the measured observables, but that the one with less degrees of freedom (I) could be uniquely excluded by future experimental constraints. This conclusion is based on the exact matching between the 4D and 5D analytical calculations of the mass spectrum – via regularizations of type I and II. Beyond a deeper insight into the Higgs peak regularizations, this matching brings another confirmation of the validity of the 5D mixed formalism. All the conclusions, deduced from regularizing the Higgs peak through a brane shift or a smoothed square profile, are expected to remain similar in realistic models with a warped extra-dimension. The complementary result of the study is that the non-commutativity disappears, both in the 4D and the 5D calculations, in the presence of higher order derivative operators. For clarity, the 4D and 5D analytical calculations, matching with each other, are presented in the first part of the paper, while the second part is devoted to the interpretation of the results.

## Introduction

The recent and historical discovery of a Higgs-like boson [1–3] around 125 GeV at the Large Hadron Collider (LHC) [4, 5] of the CERN presents the last missing piece of the particle content of the Standard Model (SM). However, even with the discovery of the Brout–Englert–Higgs scalar field [6–10], the mechanism responsible for breaking the electroweak (EW) symmetry is not fully understood; there remain some questions unresolved like, for example, determining the range of validity of the SM. If the SM is valid all the way up to the Planck scale then one can wonder why the EW energy scale (close to the Higgs mass) is so much smaller than the Planck scale. The famous Randall–Sundrum (RS) proposition of an higher-dimensional background with the Higgs boson localized on a TeV or infra-red (IR) brane [11], besides addressing the gauge hierarchy problem of Higgs mass corrections, provides an aesthetic interpretation of this apparent discrepancy between fundamental scales of nature: the measured Planck scale would be an effective four-dimensional (4D) scale whereas the gravity scale on the TeV-brane would be reduced by a warp factor down to the EW scale order (the 5D gravity scale in the bulk being still of the order of the Planck scale). The later RS version with SM fields propagating in the bulk [12] even allows one to explain the strong hierarchies among fermion masses.

At this special moment where the LHC is scrutinizing the Higgs boson properties [13–28] and exploring higher energy frontiers, it is crucial for the community to have a deep theoretical understanding of the RS paradigm, in order to develop careful phenomenological tests of such a scenario. These tests of the RS model can make use of the more and more precise experimental measurements in the Higgs sector [29–32] or of possible direct signatures from Kaluza–Klein (KK) excitations at colliders [33–48] (see Ref. [49] for a review).

Now, from the theoretical point of view, it turns out that recently there has been a debate in the literature on RS frameworks [50, 51]. A non-commutativity has appeared: different results were obtained for Higgs production/decay processes when taking $\epsilon \rightarrow 0$ and then $N_\mathrm{KK} \rightarrow \infty$ [52] or the opposite order [53]. $N_\mathrm{KK}$ is the number of exchanged excited states at the level of the loop amplitude. $\epsilon$ is the infinitesimal parameter introduced to regularize the Dirac peak along the extra-dimension associated to the Higgs scalar stuck on the IR-brane; indeed, this Higgs peak induces the so-called jump problem, for the wave functions of the fermion bulk fields, which must be regularized. It was clearly crucial for testing the Higgs sector of the RS model at LHC to shed light on those theoretical subtleties.

In this paper, we show that there exists another type of non-commutativity in a 4D calculation (based on considering gradually KK tower effects): the fermion mass spectrum expression relies on the arbitrary choice in ordering the limits $\epsilon \rightarrow 0$ and $N \rightarrow \infty$, where *N* is now the KK-index at the level of the calculation of mass eigenvalues. We point out this non-commutativity in a toy model with a brane-localized Higgs boson and fermionic matter propagating along a flat extra-dimension, but our main conclusions are expected to remain true in more realistic warped extra-dimension scenarios.

So once more, it is urgent to really understand this new non-commutativity and to determine which order of the limits on $\epsilon$, *N* has to be followed to construct a consistent model before studying its phenomenology. For that purpose, we perform calculations of the fermion mass spectrum, in both the 4D and the 5D (based on equations of motion with Yukawa terms) approaches, applying consecutively the two possible orders – assimilated to two kinds of Higgs regularization – for the above mentioned limits on $\epsilon$, *N*. Those calculations allow effectively a better insight into the Higgs peak regularization features.

This 4D calculation of the mass spectrum reveals itself to be quite ‘heavy’, due to the rich texture of the infinite fermion mass matrices, but it has the further interest to demonstrate analytically the exact matchings with the 5D calculation results. Obtaining these 4D/5D matching results represents an opportunity to confirm again the 5D formalism for KK mixings often used.

Let us specify that in order to provide various illustrations of our calculations within the above two types of regularizations, we regularize the Higgs delta peak by shifting it away from the boundary as well as smoothing it into a square profile – which constitutes an equivalent alternative.

Last but not least, we show that the non-commutativity disappears in scenarios where the high-energy (ultra-violet, UV) completion of the model leads to higher order operators with derivatives and localized at the Higgs brane.

The paper is organized accordingly to the following simple plan. While Sect. 2 is devoted to the 5D approach of the fermion mass spectrum, Sect. 3 is focused on the 4D treatment and the two calculations are compared in the synthesis made in Sect. 4. We summarize and conclude in Sect. 5.

## 5D calculations

### The model

We consider a toy model with an extra spatial dimension having a flat geometry and being parametrized by the coordinate, *y*. This extra-dimension constitutes an interval of length $\pi R$ with two boundaries at $y=0,\pi R$. The Higgs boson of the SM, embedded in a doublet under the $SU(2)_L$ gauge group, is strictly localized on the brane at $y=\pi R$ while some fermionic matter is spread out in the bulk. For illustration, let us consider the first quark generation^1^; the down-quark fields denoted by *Q* and *D* are respectively a doublet component and a singlet under $SU(2)_L$, as in the SM. The dynamics for the up-quark sector fields, $\tilde{Q}$ and *U*, is dictated by an identical Lagrangian and thus we will not repeat such an analogous analysis throughout the paper. For our task, it is sufficient to concentrate on the kinetic terms for the down-quarks as well as their Yukawa interactions, whose fundamental 5D action can be written as usual (after the EW symmetry breaking),

1 $$\begin{aligned} S_{\text {fermion}}= & {} \int \text {d}^4x~\text {d}y~\bigg [\frac{i}{2}(\bar{Q}\Gamma ^M \partial _M Q -\partial _M \bar{Q} \Gamma ^M Q ~\nonumber \\&+ \{ Q \leftrightarrow D \} )+ \delta (y-\pi R) \ (Y_5\ \bar{Q}_LHD_R\nonumber \\&+ Y^\prime _5\ \bar{Q}_RHD_L + \text {H.c.})\bigg ], \end{aligned}$$

where the index is $M=0,1,2,3,5$ and the Higgs field is developed into the 4D scalar plus its vacuum expectation value as $H=\frac{v+h(x)}{\sqrt{2}}$, *x* representing the usual four coordinates. It should be remarked that the coupling constants $Y_5$ and $Y^\prime _5$ are independent; in order to avoid the introduction of a new scale in the theory, one can choose $Y_5=yR$ and $Y^\prime _5=y'R$, where $y,y'$ are dimensionless coupling constants of $\mathcal{O}(1)$. In our notations, the 5D Dirac spinor, being the smallest irreducible representation of the Lorentz group, reads

2 $$\begin{aligned} Q = \left( \begin{array}{c} Q_L \\ Q_R \end{array}\right) \quad \text{ and } \quad D = \left( \begin{array}{c} D_L \\ D_R \end{array}\right) , \end{aligned}$$

in terms of the two two-component spinors, for the two down-quark fields.

### The KK decomposition and equations of motion

In this Sect. 2, we derive the fermion masses using the so-called exact or 5D approach. In this approach, one keeps the Yukawa mass terms that appear after EW symmetry breaking in the equations of motion for the fermion profiles along the extra-dimension [we will simply refer to those as equations of motion (EOM)]. The advantage of this approach is that the mixing among *all* KK modes for any fermion is automatically taken care of when solving the EOM. Hence this method for deriving the masses is called a 5D calculation as it incorporates the full effect of the 5D theory in the EOM.

The first step is to perform a ‘mixed’ KK decomposition of the 5D fields in Eq. (2),

3a $$\begin{aligned} Q_L&= \sum _{n=0}^{\infty } q_L^n(y)\;Q_L^n (x), \end{aligned}$$

3b $$\begin{aligned} Q_R&= \sum _{n=0}^{\infty } q_R^n(y)\;D_R^n (x), \end{aligned}$$

3c $$\begin{aligned} D_L&= \sum _{n=0}^{\infty } d_L^n(y)\;Q_L^n (x), \end{aligned}$$

3d $$\begin{aligned} D_R&= \sum _{n=0}^{\infty } d_R^n(y)\;D_R^n (x), \end{aligned}$$

where $Q_L^n(x)$, $D_R^n(x)$ are the 4D fields and $q_{L,R}^n(y)$, $d_{L,R}^n(y)$ are the corresponding wave functions along the extra-dimension. Although not essential for our calculations, we note for completeness that with this KK decomposition, the profiles satisfy the following normalization condition:

$$\begin{aligned} \int _0^{\pi R} \mathrm{d}y [\vert q_X(y)\vert ^2+\vert d_X(y)\vert ^2] =1; \quad \text{ with }\quad X=L,R. \end{aligned}$$

Through a factorization of the 4D fields, the mixed KK decomposition allows one to separate the Euler–Lagrange equations for the 5D fields into the 4D Dirac equations ($\mu =0,1,2,3$),

4 $$\begin{aligned} -i \bar{\sigma }^{\mu } \partial _{\mu } Q^n_L(x) + m~D^n_R(x)= & {} 0, \end{aligned}$$

5 $$\begin{aligned} -i \sigma ^{\mu } \partial _{\mu } D^n_R(x) + m~Q^n_L(x)= & {} 0, \end{aligned}$$

and the equations of motion for any excited fermion profile after EW symmetry breaking,

6a $$\begin{aligned} -\ m~q_L - q'_R + \delta (y-\pi R) \ \frac{v Y_5}{\sqrt{2}}~d_R ~&=~ 0, \end{aligned}$$

6b $$\begin{aligned} -\ m~q_R + q'_L + \delta (y-\pi R) \ \frac{v Y^\prime _5}{\sqrt{2}}~d_L ~&=~ 0, \end{aligned}$$

6c $$\begin{aligned} -\ m~d_L - d'_R + \delta (y-\pi R) \ \frac{v Y^\prime _5}{\sqrt{2}}~q_R ~&=~ 0, \end{aligned}$$

6d $$\begin{aligned} -\ m~d_R + d'_L + \delta (y-\pi R) \ \frac{v Y_5}{\sqrt{2}}~q_L ~&=~ 0, \end{aligned}$$

where the ‘ $'$ ’ exponent after any wave function denotes the derivative with respect to the fifth coordinate, *y*. We have assumed real Yukawa coupling constants and *m* masses for simplicity, but this kind of analysis is generalizable to cases with complex phases.

The variation of the action combined with the above EOM on the boundaries give rise either to the Dirichlet Boundary Conditions (BC) on both boundaries (i.e. vanishing profiles at the two endpoints), denoted $$(--)$$ and to be assigned to $$q_R$$ and $$d_L$$, or to the Neumann BC (vanishing derivatives), denoted $$(++)$$ and assigned to $$q_L$$ and $$d_R$$. Now, due to the $$\delta (y-\pi R)$$-term in Eq. (6a), its infinitesimal integration around $$y=\pi R$$ leads to two distinct values of $$q_R$$ at that point, which together with the unique $$q_R$$$$(--)$$ BC renders the value of this profile at $$y=\pi R$$ ambiguous: this is the ‘jump’ problem [54], first described on an interval in Ref. [55], which also arises of course for the $$d_L$$ profile in Eq. (6d).

To avoid this ambiguity one has to regularize the Higgs peak [55]: this can be done via shifting the Dirac peak away from the boundary by a small ($\epsilon R$) amount, or via smoothing the peak by giving it a narrow width (like a normalized square function of width $\epsilon R$). Then one imposes the $(--)$ BCs and solves the EOM (involving $\epsilon$) to find the fermion masses, before finally taking the limit, $\epsilon \rightarrow 0$, in order to recover the wanted brane-localized Higgs situation. We are going to realize explicitly those two schemes of $\epsilon$-regularization in the next two subsections.

### Moving the Higgs peak

If one shifts the Higgs peak by a distance $\epsilon R$ away from the $\pi R$-boundary,

7 $$\begin{aligned} \delta \left( y-\pi R\right) \rightarrow \delta \left( y-(\pi -\epsilon )R\right) , \end{aligned}$$

then profile jumps move from the boundary to the bulk. The EOM that one needs to solve become,

8a $$\begin{aligned} -\ m~q_L - q'_R + \delta \left( y-(\pi -\epsilon )R\right) \ \frac{vY_5}{\sqrt{2}}~d_R = 0, \end{aligned}$$

8b $$\begin{aligned} -\ m~q_R + q'_L + \delta \left( y-(\pi -\epsilon )R\right) \ \frac{vY^\prime _5}{\sqrt{2}}~d_L = 0,\end{aligned}$$

8c $$\begin{aligned} -\ m~d_L - d'_R + \delta \left( y-(\pi -\epsilon )R\right) \ \frac{vY^\prime _5}{\sqrt{2}}~q_R = 0, \end{aligned}$$

8d $$\begin{aligned} -\ m~d_R + d'_L + \delta \left( y-(\pi -\epsilon )R\right) \ \frac{vY_5}{\sqrt{2}}~q_L = 0. \end{aligned}$$

Solving this set of equations is not very complicated since, for $0\le y < (\pi -\epsilon )R$ and $(\pi -\epsilon )R < y \le \pi R$, the above equations become identical to the free equations, i.e. EOM without the Yukawa terms. For the $q_R$, $d_L$ solutions satisfying the $(--)$ BC and the $q_L$, $d_R$ solutions with $(++)$ BC, at $y=0$ (BCs still induced by the action variation combined with the new EOM (8a)–(8d) on the boundaries), we get the following physical profiles:

9 $$\begin{aligned} \begin{aligned}&q_L(y) = \mathcal C \cos (m y),\quad q_R(y) =-\mathcal C \sin (m y), \quad \\&d_R(y) = \mathcal C \cos (m y),\quad d_L(y) =\mathcal C \sin (m y), \end{aligned} \end{aligned}$$

which are valid for $0\le y < (\pi -\epsilon )R$. $\mathcal C$ denotes the normalization factor. For $(\pi -\epsilon )R < y \le \pi R$, we obtain the following general EOM solutions:

10 $$\begin{aligned} \begin{aligned}&\hat{q}_L(y) = B_1 \cos (m y) + B_2 \sin (m y),\quad \\&\hat{q}_R(y) = B_2 \cos (m y) - B_1 \sin (m y), \\&\hat{d}_L(y) = B_3 \cos (m y) + B_4 \sin (m y), \quad \\&\hat{d}_R(y) = B_4 \cos (m y) - B_3 \sin (m y), \end{aligned} \end{aligned}$$

where the $B_i$ are arbitrary constants that are fixed by the normalizations. From Eqs. (8a)–(8d), we see that the amount of jump that a field undergoes is proportional to the value of some other profile exactly at $y=(\pi -\epsilon )R$. Hence to connect the two sets of solutions in Eqs. (9) and (10), one needs to assign some values for these profiles at the jump point. We use the following convention for a generic profile:

11 $$\begin{aligned} f\left( (\pi -\epsilon )R\right) = \frac{1}{1+c}[f\left( (\pi -\epsilon )R\right) +c \hat{f}\left( (\pi -\epsilon )R\right) ], \nonumber \\ \end{aligned}$$

i.e. we take the weighted average of the limiting values of the function approaching from both sides, which, for $c=1$, translates into the normal averaging. The continuity conditions read then

12a $$\begin{aligned}&q_R\left( (\pi -\epsilon )R\right) - \hat{q}_R\left( (\pi -\epsilon )R\right) \nonumber \\&\quad = \frac{-vY_5}{\sqrt{2} (1+c)} [d_R\left( (\pi -\epsilon )R\right) + c\, \hat{d}_R\left( (\pi -\epsilon )R\right) ],\end{aligned}$$

12b $$\begin{aligned}&q_L\left( (\pi -\epsilon )R\right) - \hat{q}_L\left( (\pi -\epsilon )R\right) \nonumber \\&\quad = \frac{vY^\prime _5}{\sqrt{2} (1+c)} [d_L\left( (\pi -\epsilon )R\right) + c\, \hat{d}_L\left( (\pi -\epsilon )R\right) ],\end{aligned}$$

12c $$\begin{aligned}&d_R\left( (\pi -\epsilon )R\right) - \hat{d}_R\left( (\pi -\epsilon )R\right) \nonumber \\&\quad = \frac{-vY^\prime _5}{\sqrt{2} (1+c)} [q_R\left( (\pi -\epsilon )R\right) + c\, \hat{q}_R\left( (\pi -\epsilon )R\right) ],\end{aligned}$$

12d $$\begin{aligned}&d_L\left( (\pi -\epsilon )R\right) - \hat{d}_L\left( (\pi -\epsilon )R\right) \nonumber \\&\quad = \frac{vY_5}{\sqrt{2} (1+c)} [q_L\left( (\pi -\epsilon )R\right) + c\, \hat{q}_L\left( (\pi -\epsilon )R\right) ]. \end{aligned}$$

Injecting Eqs. (9)–(10) in these four relations gives us the following constant expressions:

13 $$\begin{aligned} B_1= & {} B_4\nonumber \\= & {} \frac{\mathcal C [(1 \!+\! c)^2 (X\!+\!X^\prime ) \sin \left( 2 (\pi - \epsilon ) mR \right) -2( (1\!+\! c)^2 \!+\! c XX^\prime )] }{2[c^2XX^\prime -(1\!+\!c^2)]},\nonumber \\ \end{aligned}$$

14 $$\begin{aligned} B_3= & {} -B_2\nonumber \\= & {} \frac{\mathcal C (1 + c)^2[X-X^\prime + (X+X^\prime ) \cos \left( 2 (\pi - \epsilon ) mR\right) ] }{2[c^2XX^\prime -(1+c^2)]}, \end{aligned}$$

where $X=vY_5/\sqrt{2}$ and $X^\prime = vY^\prime _5/\sqrt{2}$. One can now apply the BC for the $(--)$ modes on the $y=\pi R$ brane,

15 $$\begin{aligned} \hat{q}_R(\pi R) = \hat{d}_L(\pi R) =0. \end{aligned}$$

For small $\epsilon \rightarrow 0$, this requires

16 $$\begin{aligned} \tan \left( \pi R\; m\right) = \frac{\sqrt{2}(1+c)^2 vY_5}{2(1+c)^2+cv^2Y_5Y^\prime _5}, \end{aligned}$$

which for $c=1$ becomes

17 $$\begin{aligned} \tan \left( \pi R\; m\right)= & {} \frac{4\sqrt{2} vY_5}{8+v^2Y_5Y^\prime _5}. \end{aligned}$$

This relation gives directly the solutions for the fermion mass spectrum.

It is possible to choose another order of calculation. Indeed, one can first derive the BC for the four profiles at $y=0,\pi R$ and thus take into account their effects on the EOM terms in Eqs. 6a–6d. At this level, we can first show (as we do in Appendix A) that the usual $(--)$ and $(++)$ BC exist in the case where the EOM (6a)–(6d), containing boundary terms, hold. Now the $(--)$ BC assigned to the $d_L$, $q_R$ wave functions have the effect of eliminating the $\delta (y-\pi R)$ terms in Eqs. 6b–6c.

Then the rest of the procedure to find the mass spectrum is identical to the previous order of calculation, except of course that the terms involving the $Y^\prime _5$ coupling constant are absent. At the next step, one introduces a regularizing $\epsilon$-shift in the EOM (6a)–(6d) (without $Y^\prime _5$ terms). As above, integrating the obtained EOM leads to conditions at $y=(\pi -\epsilon )R$ which connect the *d*, *q* profiles defined in the interval, $[0,(\pi -\epsilon )R]$, with the $\hat{d}$, $\hat{q}$ profiles on, $[(\pi -\epsilon )R,\pi R]$. These are now conditions of continuity for the $d_R$, $q_L$ profiles across $y=(\pi -\epsilon )R$,

18a $$\begin{aligned} q_R\left( (\pi -\epsilon )R\right) - \hat{q}_R\left( (\pi -\epsilon )R\right) \ =&\ \frac{-vY_5}{\sqrt{2} } d_R\left( (\pi -\epsilon )R\right) , \end{aligned}$$

18b $$\begin{aligned} q_L\left( (\pi -\epsilon )R\right) - \hat{q}_L\left( (\pi -\epsilon )R\right) \ =&\ 0, \end{aligned}$$

18c $$\begin{aligned} d_R\left( (\pi -\epsilon )R\right) - \hat{d}_R\left( (\pi -\epsilon )R\right) \ =&\ 0, \end{aligned}$$

18d $$\begin{aligned} d_L\left( (\pi -\epsilon )R\right) - \hat{d}_L\left( (\pi -\epsilon )R\right) \ =&\ \frac{vY_5}{\sqrt{2}}q_L\left( (\pi -\epsilon )R\right) , \end{aligned}$$

due to the absence of $Y^\prime _5$ terms on the right-hand side of Eqs. 18b–18c. The consequence is that these profiles are well defined at $y=(\pi -\epsilon )R$, where $d_R=\hat{d}_R$, $q_L=\hat{q}_L$, which fixes uniquely the amounts of discontinuity in Eqs. (18a) and 18d; there is thus no need to choose any *c*-prescription like in Eq. (12). One continues to follow the same steps of calculation as in the previous procedure, with the same BC as well. Replacing the $d_L$, $q_R$ ($d_R$, $q_L$) profiles in Eqs. 18a–18d with their expressions dictated by the free EOM and the Dirichlet (Neumann) BC at $y=0$, as well as $\hat{d}_{L,R}$, $\hat{q}_{L,R}$ with the general expressions for free profiles, gives rise to a system whose solutions for the constants once injected in the BC, $\hat{q}_R(\pi R) = \hat{d}_L(\pi R) =0$, lead to the fermion mass spectrum,

19 $$\begin{aligned} \tan (\pi R \;m)= & {} \frac{vY_5}{\sqrt{2}}, \end{aligned}$$

in the $\epsilon \rightarrow 0$ limit. It turns out that this mass result can be obtained from Eq. (16) by setting $Y^\prime _5=0$, in which case indeed the *c*-dependence disappears.

### Smoothing the Higgs peak

We can alternatively replace the Higgs Dirac peak at the boundary by a normalized square function, of width $\epsilon R$ and height $1/\epsilon R$, so that the Dirac peak is recovered in the limit, $\epsilon \rightarrow 0$. With this smooth profile, one gets the following EOM:

20a $$\begin{aligned} -\ m~q_L - q'_R + \frac{\Theta \left( y-(\pi -\epsilon )R\right) }{\epsilon R} \ \frac{vY_5}{\sqrt{2}}~d_R = 0, \end{aligned}$$

20b $$\begin{aligned} -\ m~q_R + q'_L + \frac{\Theta \left( y-(\pi -\epsilon )R\right) }{\epsilon R} \ \frac{vY^\prime _5}{\sqrt{2} } d_L = 0,\end{aligned}$$

20c $$\begin{aligned} -\ m~d_L - d'_R + \frac{\Theta \left( y-(\pi -\epsilon )R\right) }{\epsilon R} \ \frac{vY^\prime _5}{\sqrt{2} }~q_R = 0, \end{aligned}$$

20d $$\begin{aligned} -\ m~d_R + d'_L + \frac{\Theta \left( y-(\pi -\epsilon )R\right) }{\epsilon R} \ \frac{vY_5}{\sqrt{2} }~q_L = 0, \end{aligned}$$

where $\Theta (y) =1$ for $y\ge 0$ and zero otherwise. In the range $0\le y < (\pi -\epsilon )R$, these equations correspond to the free EOM and have the same solutions as in Eq. (9) if we impose once more the $(--)$ and $(++)$ BCs at $y=0$. Assuming $Y_5=Y^\prime _5$ for simplicity, the following generic ansatz solves the EOM (20a)–(20d) in the range $(\pi -\epsilon )R \le y \le \pi R$:

21 $$\begin{aligned} \hat{f}_X(y)= & {} A_{f_X} \exp \left( \sqrt{\frac{v^2 Y_5^2 - 2 m^2 \epsilon ^2 R^2}{2 \epsilon ^2 R^2}}y\right) \nonumber \\&+ B_{f_X} \exp \left( -\sqrt{\frac{v^2 Y_5^2 - 2 m^2 \epsilon ^2 R^2}{2 \epsilon ^2 R^2}}y\right) , \end{aligned}$$

$f_X$ standing for any profile and $A_{f_X}$, $B_{f_X}$ being normalization constants. Demanding that all the profiles are continuous across $y=\left( \pi -\epsilon \right) R$ and setting $\hat{q}_R(\pi R) = \hat{d}_L(\pi R) = 0$ (BC for the $(--)$ modes) gives us the following condition on the mass:

22 $$\begin{aligned} \tan \left( \pi R \; m\right)= & {} \sqrt{\frac{vY_5 - \sqrt{2}m\epsilon R}{vY_5 + \sqrt{2}m\epsilon R}} \;\tanh \left( \sqrt{\frac{v^2Y_5^2 - 2m^2\epsilon ^2 R^2}{2}}\right) .\nonumber \\ \end{aligned}$$

In the limit $\epsilon \rightarrow 0$ this simplifies to

23 $$\begin{aligned} \tan \left( \pi R \;m\right)= & {} \tanh \left( \sqrt{\frac{v^2Y_5^2}{2}}\right) . \end{aligned}$$

As in the case of the shifted delta function, if one first imposes instead the BC for the $(--)$ modes, $q_R(\pi R) = d_L(\pi R) =0$, the Yukawa terms in Eqs. 6b–6c are eliminated. Then solving the EOM (20a)–(20d) with an $\epsilon R$-square function but without those two Yukawa terms, one recovers, through the same steps of calculation, the simple mass spectrum of Eq. 19.

## 4D calculations

### The KK decomposition and mass matrices

In this Sect. 3, considering the same model as the one defined by the Lagrangian (1), we calculate the fermion masses in the maybe more intuitive approach referred to as the perturbative or 4D calculation. To obtain the fermion profiles, here, one considers the free EOM, i.e. the equations without Yukawa mass terms. As a result, unlike the 5D point of view addressed in the previous Sect. 2, one needs to diagonalize the fermion mass matrices to include the whole KK mass mixing effect. The 4D approach denomination relies on the fact that one starts from a 4D model without KK modes and the entire KK tower is taken into account gradually, through the limit $N\rightarrow \infty$. It is also called a perturbative approach in the sense that the Yukawa interaction is incorporated via infinite series terms.

Now, these infinite numbers of KK excitations lead to infinite-dimensional mass matrices whose exact diagonalization can represent a challenging task. However, in certain cases it is possible analytically as we shall illustrate in Sect. 3. The aim being to compare the fermion masses obtained by diagonalizing the complete mass matrix with the ones obtained from the previous 5D approach.

We start by decomposing the 5D fields in KK towers like,

24a $$\begin{aligned} Q_L&= \sum _{n=0}^{\infty } q_L^n(y)\;Q_L^n (x), \end{aligned}$$

24b $$\begin{aligned} Q_R&= \sum _{n=0}^{\infty } q_R^n(y)\;Q_R^n (x), \end{aligned}$$

24c $$\begin{aligned} D_L&= \sum _{n=0}^{\infty } d_L^n(y)\;D_L^n (x), \end{aligned}$$

24d $$\begin{aligned} D_R&= \sum _{n=0}^{\infty } d_R^n(y)\;D_R^n (x), \end{aligned}$$

which gives rise to the following KK mass terms in the 4D effective Lagrangian:

$$\begin{aligned} \mathcal L_\mathrm{KK}= & {} -\sum _{n=0}^\infty [M_{qn} \bar{Q}_L^n(x) Q_R^n(x)\\&+M_{dn} \bar{D}_L^n(x) D_R^n(x)] + \mathrm{H.c.} \end{aligned}$$

where

25 $$\begin{aligned} M_{qn} = M_{dn} = \frac{n}{R}. \end{aligned}$$

The complete quark mass matrix in the 4D effective picture, after EW symmetry breaking, reads

$$\begin{aligned} \mathcal L_\mathrm{mass} = -\bar{\Psi }_L\,\cdot \,\left[ M\right] \,\cdot \Psi _R + \mathrm{H.c.} \end{aligned}$$

and can be expressed, in the ‘combined’ basis for the left- and right-handed fields,

26 $$\begin{aligned} \Psi ^t_L= & {} (Q_L^0,D_L^0,Q_L^1,D_L^1,Q_L^2,D_L^2,\ldots ), \nonumber \\ \Psi ^t_R= & {} (Q_R^0,D_R^0,Q_R^1,D_R^1,Q_R^2,D_R^2,\ldots ), \end{aligned}$$

by the following infinite matrix:

27 $$\begin{aligned} \left[ M\right]\equiv & {} \left( \begin{array}{c@{\quad }c@{\quad }c@{\quad }c@{\quad }c@{\quad }c@{\quad }c} M_{q0} &{} \alpha _{00} &{} 0 &{} \alpha _{01 } &{} 0 &{} \alpha _{02} &{} \cdots \\ \beta _{00} &{} M_{d0} &{} \beta _{01} &{} 0 &{} \beta _{02} &{} 0 &{} \cdots \\ 0 &{} \alpha _{10} &{} M_{q1} &{} \alpha _{11} &{} 0 &{} \alpha _{12} &{} \cdots \\ \beta _{10} &{} 0 &{} \beta _{11} &{} M_{d1} &{} \beta _{12} &{} 0 &{} \cdots \\ 0 &{} \alpha _{20} &{} 0 &{} \alpha _{21} &{} M_{q2} &{} \alpha _{22} &{} \cdots \\ \beta _{20} &{} 0 &{} \beta _{21} &{} 0 &{} \beta _{22} &{} M_{d2} &{} \cdots \\ \vdots &{} \vdots &{} \vdots &{} \vdots &{} \vdots &{} \vdots &{} \ddots \end{array} \right) , \nonumber \\ \end{aligned}$$

with

28 $$\begin{aligned} \alpha _{ij}= & {} Y_5\int _0^{\pi R} \mathrm{d}y \ \delta (y-\pi R) \ \frac{v}{\sqrt{2}} \ q_L^i (y) \ d_R^j(y),\end{aligned}$$

29 $$\begin{aligned} \beta _{ij}= & {} Y^\prime _5\int _0^{\pi R} \mathrm{d}y \ \delta (y-\pi R) \ \frac{v}{\sqrt{2}} \ d_L^i (y) \ q_R^j(y). \end{aligned}$$

To try to match the different regularizations performed in the 5D approach of Sect. 2, we will treat similarly the Higgs peak – by either moving or smoothing it – in the 4D calculations of next two subsections.

### Moving the Higgs peak

The fields (26) undergo the unitary transformation matrices to the physical basis and the squared modulus of the quark masses, $\vert m \vert ^2$, are the eigenvalues, noted $\lambda$, of the infinite-dimensional matrix, $[M^\dag M]$. For a general Higgs profile, we present in Appendix B one of the main results of the paper: the characteristic equation (CE), for the infinite $[M^\dag M]$ matrix, whose solutions are the eigenvalues, $\lambda = \vert m \vert ^2$. From the obtained expression of the CE terms shown there, a logical structure in series emerges for such a general case. The CE contains infinite series of various types which can be written using the generic structures, $A_n$ and $B_n$, involving, respectively, $\alpha _{ij}$ and $\beta _{ij}$.

Let us now focus on the case of a Higgs peak infinitesimally shifted at some point, $y=(\pi -\epsilon ) R$, along the extra-dimension as in Eq. (7). Then the CE takes a much simpler form since the functions, $\alpha _{ij}$ and $\beta _{ij}$, are factorizable in *i* and *j*,

30 $$\begin{aligned} \alpha _{ij}= & {} \frac{vY_5}{\sqrt{2}}\, q_L^i ((\pi -\epsilon )R) \times d_R^j((\pi -\epsilon )R), \end{aligned}$$

31 $$\begin{aligned} \beta _{ji}= & {} \frac{vY^\prime _5}{\sqrt{2}}\, q_R^i ((\pi -\epsilon )R) \times d_L^j((\pi -\epsilon )R), \end{aligned}$$

so that accordingly to Eq. (B.3)

32 $$\begin{aligned} A_{n>1} = B_{n>1} =0, \end{aligned}$$

due to the anti-symmetric constructions of $A_n$ and $B_n$. As a result the generic CE of Eq. (B.1) simplifies to

33 $$\begin{aligned}&1 + \sum _{q_1;d_1}(-\lambda )\frac{(\alpha _{q_1d_1})^2 +(\beta _{d_1q_1})^2}{(M_{q_1}^2-\lambda )(M_{d_1}^2-\lambda )}\nonumber \\&\quad +\sum _{q_1,q_2;d_1,d_2}(-\lambda )^2 \frac{(\alpha _{q_1d_1})^2(\beta _{d_2q_2})^2}{(M_{q_1}^2-\lambda )(M_{d_1}^2-\lambda )(M_{q_2}^2-\lambda )(M_{d_2}^2-\lambda )}\nonumber \\&\quad \times \left( 1-\delta _{q_1q_2}\frac{M_{q_2}^2}{\lambda }\right) \left( 1-\delta _{d_1d_2}\frac{M_{d_2}^2}{\lambda }\right) \nonumber \\&\quad -\sum _{Q_1;D_1} 2 \ M_{Q_1}M_{D_1} \frac{\alpha _{Q_1D_1} \beta _{D_1Q_1}}{(M_{Q_1}^2-\lambda )(M_{D_1}^2-\lambda )}\nonumber \\&\quad +\sum _{Q_1<Q_2:d_1,d_2} \frac{2 (-\lambda ) M_{Q_1}M_{Q_2}}{(M_{Q_1}^2-\lambda )(M_{Q_2}^2-\lambda )}\nonumber \\&\quad \times \frac{\alpha _{Q_1d_1}\alpha _{Q_2d_1} \beta _{d_2Q_1}\beta _{d_2Q_2}}{(M_{d_1}^2-\lambda )(M_{d_2}^2-\lambda )}\times \left( 1-\delta _{d_1d_2}\frac{M_{d_2}^2}{\lambda }\right) \nonumber \\&\quad +\sum _{q_1,q_2:D_1<D_2} \frac{2 (-\lambda ) M_{D_1}M_{D_2}}{(M_{D_1}^2-\lambda )(M_{D_2}^2-\lambda )} \!\times \!\frac{\alpha _{q_1D_1}\alpha _{q_1D_2} \beta _{D_1q_2}\beta _{D_2q_2}}{(M_{q_1}^2-\lambda )(M_{q_2}^2-\lambda )}\nonumber \\&\quad \times \left( 1-\delta _{q_1q_2}\frac{M_{q_2}^2}{\lambda }\right) \nonumber \\&\quad +\sum _{Q_1<Q_2;D_1<D_2} 2\left( \prod _{i=1,2}\frac{ M_{Q_i}M_{D_i}}{(M_{Q_i}^2-\lambda )(M_{D_i}^2-\lambda )}\right) \nonumber \\& \quad \times (\alpha _{Q_1D_1}\alpha _{Q_2D_2}\times \beta _{D_1Q_1}\beta _{D_2Q_2}\nonumber \\&\quad + \alpha _{Q_1D_2}\alpha _{Q_2D_1}\times \beta _{D_2Q_1}\beta _{D_1Q_2}) = 0. \end{aligned}$$

Here and elsewhere, unless specified otherwise, a sum over any index is assumed to be running from 0 to $\infty$; in the above relation, the KK masses obey e.g., $M_{q_1} = q_1/R$ where $q_1$ is a running integer (slightly different writing from Eq. (25) to ease notations). We stress that to derive Eq. (33), no approximation has been made, or in other words this equation exhibits the complete CE in this case. Choosing the $(--)$ and $(++)$ BCs (from the action variation and free EOM on boundaries) for the quark profiles, to end up with a chiral theory, we get the following normalized solutions of the free EOM^2^:

34 $$\begin{aligned}&q^n_L(y) = d^n_R(y)=\sqrt{\frac{2}{\pi R}}\cos \left( \frac{ny}{R}\right) ,\quad \nonumber \\&\quad -q^n_R(y) = d^n_L(y)=\sqrt{\frac{2}{\pi R}}\sin \left( \frac{ny}{R}\right) \quad \text {for} \quad n >0 \nonumber \\&q^0_L(y) = d^0_R(y)=\sqrt{\frac{1}{\pi R}},\quad \nonumber \\&\quad -q^0_R(y) = d^0_L(y)=0 \quad \quad \text {for} \quad n =0. \end{aligned}$$

With these solutions, the $\alpha _{ij}$ and $\beta _{ji}$ functions of Eqs. (30)–(31) become

35 $$\begin{aligned} \alpha _{ij}= & {} \frac{\sqrt{2} vY_5}{\pi R}\, \cos (i(\pi -\epsilon )) \cos (j(\pi - \epsilon )),\quad \alpha _{00}=\frac{v Y_5}{\sqrt{2} \pi R},\nonumber \\\end{aligned}$$

36 $$\begin{aligned} \beta _{ji}= & {} \frac{- \sqrt{2} vY^\prime _5}{\pi R}\, \sin (i(\pi -\epsilon )) \sin (j(\pi -\epsilon )),\nonumber \\&\quad \beta _{j0}=\beta _{0i}=\beta _{00}=0. \end{aligned}$$

We are now in possession of all the necessary tools to simplify and solve the CE (33) in terms of the mass. Computing analytically all the involved infinite sums (over KK modes), we find the following compact form for the CE, in the final limit $\epsilon \rightarrow 0$:

37 $$\begin{aligned}&1 + \frac{1}{4} v^2Y_5Y^\prime _5 + \frac{1}{64} v^4(Y_5Y^\prime _5)^2 = \frac{v^2Y_5^2}{2} \cot ^2{(\pi R \sqrt{\lambda })}, \end{aligned}$$

38 $$\begin{aligned}&\mathrm{or}\quad \tan ^2(\pi R\sqrt{\vert m \vert ^2}) =\left( \frac{4\sqrt{2} vY_5}{8+v^2Y_5Y^\prime _5}\right) ^2 . \end{aligned}$$

Let us add a few comments, for the reader, about the methods used to derive that result. The term on the right-hand side of Eq. 37 comes from $(++)$ mode contributions only, in the sense that it follows from the series of Eq. (33) (second term of the whole expression):

39 $$\begin{aligned} \sum _{q_1;d_1}(-\lambda )\frac{(\alpha _{q_1d_1})^2}{(M_{q_1}^2-\lambda )(M_{d_1}^2-\lambda )}, \end{aligned}$$

if one invokes the following identity:

$$\begin{aligned} \sum _{n=0}^\infty \frac{1}{n^2-x^2} = -\frac{1}{2x^2}[1+\left( \pi x\right) \cot \left( \pi x\right) ], \end{aligned}$$

where *x* is some function of *R* and $\lambda$. All the other terms of Eq. (33), except the fifth one (last term of the second line) and the last one (two last lines), do not give contributions in the limit $\epsilon \rightarrow 0$. The non-vanishing terms of Eq. (33) can be re-expressed as combinations of the (Hurwitz) Lerch transcendent,^3^

$$\begin{aligned} \Phi (e^{i\epsilon },1,x)= & {} \sum _{n=0}^\infty \frac{e^{in\epsilon }}{n+x}\\= & {} -\gamma - \psi \left( x\right) - \log (-i\epsilon ) + \mathcal O(\epsilon ), \end{aligned}$$

where $\gamma$ is the Euler–Mascheroni constant and $\psi \left( x\right) = \Gamma ^\prime (x)/\Gamma (x)$ is the so-called digamma function (logarithmic derivative of the gamma function).

At this stage, we insist on the fact that in order to obtain Eq. (38) we have first written the CE of the mass matrix in Eq. (33) and calculated its KK summations up to $N\rightarrow \infty$, *before* imposing the limit $\epsilon \rightarrow 0$ on the obtained CE – as a last step. If, however, it is realized in the opposite order, i.e. first applying $\epsilon \rightarrow 0$ on the mass matrix (27) (so that the matrix elements $\beta _{ij}\rightarrow 0$ since $q^n_R(\pi R) = d^n_L(\pi R)=0$), *before* writing the matrix CE and working out its infinite KK sums or in other words taking its limit for $N\rightarrow \infty$ (without $\beta _{ij}$ series anymore), one would obtain

40 $$\begin{aligned} \tan ^2(\pi R\sqrt{\vert m \vert ^2})= & {} \left( \frac{vY_5}{\sqrt{2}}\right) ^2, \end{aligned}$$

instead of Eq. (38). Equation (40) originates solely from the series in Eq. (39). As already observed in the 5D approach, one would obtain the same result as in Eq. (40) by setting $Y^\prime _5=0$ in Eq. (38); this is logical since the $\beta _{ij}$ are proportional to $Y'_5$.

**Table 1:**
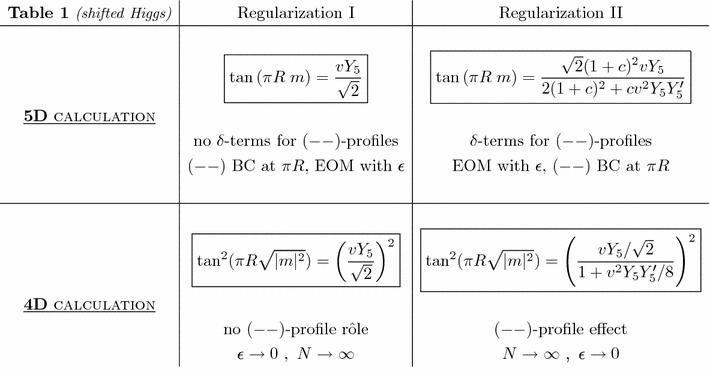
Quark mass spectrum for a shifted Higgs peak

### Smoothing the Higgs peak

Alternatively, the Higgs Dirac peak at the boundary can be replaced by a normalized square function, of width $\epsilon R$, as in Eqs. (20a)–(20d). Then, using the profiles from Eq. (34), we see that, for $i,j>0$,

$$\begin{aligned} \alpha _{ij}= & {} \frac{vY_5}{\sqrt{2}\epsilon R} \ \int _{(\pi -\epsilon )R}^{\pi R} \mathrm{d}y \; q_L^i (y) \ d_R^j(y)\\ \!= & {} \! \frac{-vY_5}{\sqrt{2}\epsilon R}\left( \frac{\sin [(i\!+\!j)(\pi \!-\!\epsilon )]}{i+j}\!+\!\frac{\sin [(i-j)(\pi -\epsilon )]}{i-j}\right) , \\ \beta _{ji}= & {} \frac{vY^\prime _5}{\sqrt{2}\epsilon R} \int _{(\pi -\epsilon )R}^{\pi R} \mathrm{d}y\; d_L^j (y) \ q_R^i(y)\\= & {} \frac{-vY'_5}{\sqrt{2}\epsilon R}\left( \frac{\sin [(i\!+\!j)(\pi -\epsilon )]}{i\!+\!j}\!-\!\frac{\sin [(i\!-\!j)(\pi \!-\!\epsilon )]}{i\!-\!j}\right) , \end{aligned}$$

which means that the functions $\alpha _{ij}$ and $\beta _{ji}$ are no longer factorizable in *i*, *j* – so that the simplification relation (B.3) does not hold anymore. As a result, the CE of Eqs. (B.1)–(B.7), for the infinite $[M^\dag M]$ matrix, contains multiple infinite series which render difficult its simplification. Now in the absence of a compact form, like the one in Eq. (33), it is tricky to solve the CE and work out the exact squared mass eigenvalues, $\lambda = \vert m \vert ^2$.

## Interpretation of the analytical results

### A non-commutativity in the 4D approach

After having presented our analytical results, we now discuss their impacts, one by one. First, we have found that the 4D calculation gives rise to different fermion mass spectrum definitions in the two orderings of the calculation: first taking the limit $\epsilon \rightarrow 0$ (Higgs localization) in the mass matrix (27) before writing the CE and applying the limit $N \rightarrow \infty$ (here *N* refers generically to the various indices used in previous section for the KK summations), leads to the CE (40),^4^ while the inverse order of taking $N \rightarrow \infty$ in the CE and $\epsilon \rightarrow 0$ in a second step, results in Eq. (38) – in the case of an Higgs profile regularized by a shifted Dirac peak where the CE can be derived analytically from the 4D point of view (dealing with infinite mass matrices). In the former order, the fermion $(--)$ wave functions play absolutely no rôle in the calculation since the $\beta _{ij}$ off-diagonal terms of the matrix (27) vanish at the first step ($\epsilon \rightarrow 0$ limit). In contrast, the infinite KK sum over these vanishing terms gives rise non-trivially to an additional contribution in Eq. (38) which is proportional to the $Y_5'$ coupling (entering the $\beta _{ij}$). All this is summarized in the 4D line of Table 1.

This non-commutativity will be confirmed in Sect. 4.2 in the following sense: we will see that these two 4D calculation orderings correspond to two different 5D calculations.

In the context with bulk fermions coupled to a brane Higgs, the non-commutativity pointed out here – the difference between the two orderings of the limits $\epsilon \rightarrow 0$ and $N \rightarrow \infty$ – differs from the non-commutativity discussed in the literature [50, 51] (in the RS framework)^5^: the latter one concerns the different results obtained from taking first $\epsilon \rightarrow 0$ and then $N_\mathrm{KK} \rightarrow \infty$ as in Ref. [52], or the opposite order as in Ref. [53]. Here, $N_\mathrm{KK}$ denotes the number of exchanged excited modes included at the level of the one-loop amplitude, when calculating the gluon–gluon fusion mechanism or the Higgs decay rate into two photons (the loop momentum integration is performed at the really first step).

While the 4D order $\epsilon \rightarrow 0$, $N_\mathrm{KK} \rightarrow \infty$ matches the 5D calculation (avoiding the very notion of KK state) with a Higgs strictly stuck on the TeV-brane (where the $(--)$ KK modes vanish) [50], the opposite 4D order – with the brane-limit taken only at last – renders the Higgs sensitive to $(--)$ KK states and thus corresponds to the 5D approach with a narrow bulk-Higgs field localized toward the brane [50] (unsuppressed ‘resonance contribution’ from high-mass KK states which can resolve the Higgs wave function [60]). It was also found in Ref. [50] that the limit $\epsilon \rightarrow 0$, for the Higgs profile regulator, can be taken either before or after performing loop integrations.

The question^6^ about the non-commutativity of $\epsilon \rightarrow 0$ and $N_\mathrm{KK} \rightarrow \infty$ has a formal interest and was discussed for technical reasons since one has to impose anyway a $\Lambda$ cut-off at the end of the day, due to the non-renormalizability of higher-dimensional theories (or their induced low gravity scale), so that $N_\mathrm{KK}$ is bounded from above. It was found in Refs. [50, 51] that once the loop calculation is performed in a realistic context with a consistent UV regulator such as dimensional regularization (or with a hard UV momentum cut-off on the 4D loop integral), the non-commutativity ambiguity disappears. The present non-commutativity of $\epsilon \rightarrow 0$ and $N \rightarrow \infty$ raises a new question, because the $\Lambda$ cut-off must not be applied on *N* (see Sect. 4.4). This physical question about the interpretation of the non-commutativity will be addressed in Sect. 4.3.

### Matching the 4D and 5D approaches

In the 5D approach, there are also two possible ways for calculating the fermion mass spectrum, as described in Sect. 2 and summarized in the 5D line of Table 1.

In one way, the BC at $\pi R$ is imposed for the $(--)$ profiles in a first stage so that the two terms in Eqs. (6b)–(6c) involving both the $\delta (y-\pi R)$ peak and a $(--)$ profile, $d_L(y)$ or $q_R(y)$, vanish (after integration). In a second stage, one solves the EOM system (6a)–(6d) with a regularized Higgs peak, e.g. shifted by an amount $\epsilon R$.

The other way consists of first solving the system (8a)–(8d) with an $\epsilon R$-shifted Higgs, so that the terms in Eqs. (8b)–(8c) involving both $\delta (y-(\pi -\epsilon ) R)$ and a $(--)$ profile, $d_L$ or $q_R$, really contribute. Then one imposes the BC at $\pi R$ for the $(--)$ profiles, which does not eliminate the above terms. The mass spectrum is dictated by those last conditions.

Those two calculation orderings result in two different mass spectrum definition given by Eqs. (16) and (19), which are copied in the 5D line of Table 1; the angle of the tangent function is only defined modulo $n\pi$, which gives rise to the KK eigen-mass tower $m_n$ ($n\in \mathbb {N}$ as in Eqs. (3a)–(3d)). The effect of the EW symmetry breaking is thus a shift of $\arctan (vY_5/\sqrt{2})/\pi R$ in the KK mass tower *n* / *R*, for the case of the left column in Table 1.

As expected,^7^ there is a mass spectrum matching between the 4D and 5D calculations that Table 1 exhibits. Although expected, this matching was not trivial to demonstrate analytically, especially due to the complexity of dealing with the infinite 4D mass matrix (27). Furthermore, it turns out that there are in fact two distinct 4D/5D matchings, for the two calculation orders performed in 4D (cf. Sect. 4.1) and 5D (described in previous paragraph) that we thus commonly denote in the table as regularizations of type I and II – see the discussion in Sect. 4.3. The 4D/5D matching in the regularization of type I is explicit: the two equations obtained give rise to the same possible mass spectra. In the regularization of type II, the 4D/5D matching occurs exactly for $c=1$ as show the two mass equations; it means that other 5D *c*-prescriptions (i.e. $c\ne 1$) should not represent experimentally distinct regularizations^8^ (as distinct 4D approaches matching $c\ne 1$ do not exist).

The first implication of those two 4D/5D matchings is the existence of two different 4D calculations (confirming Sect. 4.1) since there are two ways of calculating the mass spectrum from the 5D point of view as well. These two ways of calculating (regularizations I and II) differ in their brane-Higgs sensitivity to the tower of bulk $(--)$ profiles; this can be described remarkably in both the 4D and the 5D approaches. From the 5D point of view, in regularization II the terms in Eqs. (8b)–(8c) coupling the VEV to $(--)$ profiles are not vanishing – in contrast with case I – as explained at the beginning of this subsection. Regarding the 4D treatment, in regularization II there is a non-vanishing contribution from the $\beta _{ij}$ terms (cf. Eq. (29)) which represent overlaps between the Higgs and $(--)$ profiles, whereas their contribution is absent in case I as discussed at the beginning of Sect. 4.1.

There is a second consequence; the two 4D/5D matchings guarantee that the 5D mixed formalism (cf. Eqs. (3a)–(3d)), followed usually in the literature, represents a correct procedure to take into account mixing effects between all KK levels which are otherwise *explicitly* included via the off-diagonal elements of the 4D mass matrix (27)).

Finally, the 4D/5D matching confirms that there exist two approaches for deriving the same mass spectrum and that in the 4D approach there is no inconsistency induced by the Higgs localization that should be regularized (as the so-called jump problem in the 5D approach). This can be interpreted by the fact that the exact 4D calculation proceeds by construction through a limit ($N\rightarrow \infty$) to obtain ‘softly’ the fermion mass expressions in the wanted higher-dimensional scenario. This limit acts typically as the regularizing limit $\epsilon \rightarrow 0$ corresponding to a brane Higgs, in the 5D framework.

The obtained 4D/5D matching also constitutes an additional confirmation of the validity of the field theory regularization usually applied in the 5D calculation (within this context of brane-localized Higgs scenarios), and leads to a global coherent picture. Now of course, to determine whether such a paradigm – relying on mathematical regularizations of an ill-defined peaked field – corresponds really to the physical model, one would have to confront it with experimental results.^9^

### On the two types of regularizations

It is mentioned at the end of Appendix C.2 in Ref. [55] (where description is limited to the simpler case $Y_5=Y_5'$) that the regularizations, called I and II here, give at most two different interpretations of the $Y_5v(=yvR)$ parameter combination (proportional to $M_DL$ in notations of Ref. [55]). Let us discuss here this twofold feature more precisely. In fact, the two types of equations in Table 1 (both similar in 4D and 5D for $c=1$) corresponding to the two regularizations constitute two different relations between the $Y^{(\prime )}_5$, *v*, *R* parameters and the physical mass solutions represented by *m*. A physical mass *m* having a unique value (the measured one), the difference between these two relations has to be either compensated by different values for $Y_5$, *v*, *R* (which do not constitute observables) in cases I and II, or canceled by setting $Y'_5$ to zero (then $Y_5$, *v*, *R* can be identical in cases I and II). There exist thus two numerically equivalent definitions of the mass value *m* so that regularizations I and II are experimentally equivalent^10^ or even strictly identical (for vanishing $Y'_5$).

Indeed, concretely, today there exist two different sets of $Y_5$, *v*, *R* values (for $Y'_5\ne 0$) reproducing the measured values of the observed fermion masses through the two definitions, $f^\mathrm{I}_{n}$ and $f^\mathrm{II}_{n}$ (solutions from the two mass equations in Table 1), associated to regularizations I and II:

41 $$\begin{aligned}&\text{ Regularization } \text{ I } \ \left\{ \begin{array}{l} m_{n}=f^\mathrm{I}_{n}(R,v,Y_5) \\ \tilde{m}_{n}=f^\mathrm{I}_{n}(R,v,\tilde{Y}_5) \end{array} \right. \nonumber \\&\text{ Regularization } \text{ II } \ \left\{ \begin{array}{l} m_{n}=f^\mathrm{II}_{n}(R,v,Y_5,Y'_5) \\ \tilde{m}_{n}=f^\mathrm{II}_{n}(R,v,\tilde{Y}_5,\tilde{Y}'_5) \end{array} \right. \end{aligned}$$

In other words, the two systems in Eq. (41) have solutions in terms of $Y^{(\prime )}_5$, *v*, *R* for the first mass eigenvalue [$m_{n=0}$] and this is true including quarks/leptons (the same formalism as here, introducing parameters $m_{\ell n}$, $Y_{\ell 5}$, $Y'_{\ell 5}$) of down or up $\mathrm{SU(2)_L}$-isospin (notations trivially extended to $\tilde{m}_{n}$, $\tilde{Y}_5$, $\tilde{Y}'_5$, $\tilde{m}_{\ell n}$, $\tilde{Y}_{\ell 5}$, $\tilde{Y}'_{\ell 5}$) from the three generations (notations to be completed with flavor indices). The fact that there exist solutions to the systems of type (41) is also due to the individual dependences of the masses on the Yukawa parameters^11^ and the higher number of $Y_5$-like parameters compared to the number of measured fermion masses. As for an overview of the other parameters, typically, the EW precision tests from the LEP collider would bound from above the *R* radius (imposing large KK masses to avoid dangerous corrections to the SM predictions for EW observables) while in the gauge boson sector $m_Z$, $m_W$, $G_F$ would allow to determine the values of the bare parameters *v*, *g*, $g'$ (through loop calculations as described e.g. in Ref. [32]), the recently measured Higgs mass fixing the quartic coupling $\lambda$ [1–3].^12^

The experimental equivalence of regularizations I and II is based on generic arguments and thus also applies to amplitudes induced by flavor changing neutral current (FCNC) effects. This leads to remarks on the FC Higgs couplings coming from misalignments between fermion masses and Yukawa couplings, in the RS framework with a brane Higgs [54]. This misalignment is quantified by a non-universal shift estimated to be, using notations of Ref. [54] except for down-quark Yukawa parameters:

42 $$\begin{aligned}&\text{ Regularization } \text{ I } \bigg \{ \Delta ^d = 0 + \Delta ^d_2 = m_d \vert m_d\vert ^2 R^{\prime 2}\nonumber \\&\quad \times \bigg ( \frac{F(c_q)}{f(c_q)^2}+\frac{F(-c_d)}{f(-c_d)^2} \bigg )\nonumber \\&\text{ Regularization } \text{ II } \bigg \{ \Delta ^d = \Delta ^d_1 + \Delta ^d_2 = m_d \vert m_d\vert ^2 R^{\prime 2} \nonumber \\&\quad \times \bigg ( \frac{2}{3} \frac{Y'_5}{Y_5} \frac{1}{f(c_q)^2f(-c_d)^2} + \frac{F(c_q)}{f(c_q)^2}+\frac{F(-c_d)}{f(-c_d)^2} \bigg ) \end{aligned}$$

where $F(c_q) = (2c_q-1)/(2c_q+1)$ and $\Delta ^d_1=0$ in case I due to vanishing contributions from $Y'_5$ terms. Note that in these equations the physical condition to reproduce the (approximated) $m_d$ mass has been used to fix the *v* parameter. Equation (42) shows that there exist two sets of parameters^13^ giving rise to the same value of $\Delta ^d$ within regularizations I (without terms proportional to $Y'_5$, as included in Ref. [54]) and II (with such terms) so that these regularizations can be experimentally equivalent. There even exist such parameters (e.g. $f(c_q) \sim 1$, $f(-c_d)\ll 1$) for $Y'_5$ and $Y_5$ of the same order of magnitude as might be wanted to not introduce new energy scales [54]. Notice that with more constraints on parameters from new experimental data and under the strong physical assumption $Y'_5 \simeq Y_5$, it could happen that the two sets of input parameters in regularizations I and II cannot reproduce the same value of $\Delta ^d$: then precise FCNC data should be used to select the correct theoretical regularization by pinning down the real and unique $\Delta ^d$ value. This experimental test is similar to the one discussed right below.

In the future, the upgraded 13 TeV LHC and other colliders will certainly provide more data. One can expect more precise measurements of the Yukawa and *hVV* [$V=Z,W$] couplings (being functions of *g*, $g'$, *v* [1–3] and *R* due to KK gauge boson mixings) or even the detection of Higgs pair production that would give information on the *hVV*, *hhVV*, *hhh* couplings (in turn on combinations of $\lambda$, *g*, $g'$, *v*, *R*). The systems of Eq. (41) would thus have to be extended to include in particular the physical $Y_{nm}$, $\tilde{Y}_{nm}$ Yukawa couplings which depend on the same parameters $Y^{(\prime )}_5$, $\tilde{Y}^{(\prime )}_5$, *v*, *R*:

43 $$\begin{aligned}&\text{ Regularization } \text{ I } \left\{ \begin{array}{l} m_{n}=f^\mathrm{I}_{n}(R,v,Y_5) \\ \tilde{m}_{n}=f^\mathrm{I}_{n}(R,v,\tilde{Y}_5) \\ Y_{nm}=g^\mathrm{I}_{nm}(R,v,Y_5) \\ \tilde{Y}_{nm}=g^\mathrm{I}_{nm}(R,v,\tilde{Y}_5) \end{array} \right. \nonumber \\&\text{ Regularization } \text{ II } \ \left\{ \begin{array}{l} m_{n}=f^\mathrm{II}_{n}(R,v,Y_5,Y'_5) \\ \tilde{m}_{n}=f^\mathrm{II}_{n}(R,v,\tilde{Y}_5,\tilde{Y}'_5) \\ Y_{nm}=g^\mathrm{II}_{nm}(R,v,Y_5,Y'_5) \\ \tilde{Y}_{nm}=g^\mathrm{II}_{nm}(R,v,\tilde{Y}_5,\tilde{Y}'_5) \end{array} \right. \end{aligned}$$

Those couplings are involved in the action terms $Y_{nm}h(x)\bar{Q}^n_L(x)D^m_R(x)$ and $\tilde{Y}_{nm} h(x)\bar{\tilde{Q}}^n_L(x)U^m_R(x)$ expressed with 4D fields representing mass eigenstates.^14^ KK mode discoveries would also add new entries (like $m_n$ with $n>0$) for the systems in Eq. (43).

With such new data coming it could happen at some point that there exist no more set of parameters satisfying one of the two types of system in Eq. (43) (more physical constraints without new degrees of freedom). This would mean that the associated regularization is ruled out by experimental data. This uniquely ruled out regularization could only correspond to the system with less parameters: regularization I (no $Y'_5$, $\tilde{Y}'_5$ parameters), since regularization II for $Y'_5,\tilde{Y}'_5\rightarrow 0$ gives back regularization I so that excluding regularization II would also exclude regularization I. In a situation of this kind where regularization I only is experimentally ruled out, regularizations I and II would obviously not be experimentally equivalent.

Let us simply remark here that it is not trivial to conclude *intuitively* on the experimental equivalence of the two regularizations. Indeed in regularization II, from the 4D point of view, first taking $N \rightarrow \infty$ leads to have in a first step a full 5D theory with complete (i.e. infinite) 5D field KK decompositions. Then imposing the $\epsilon \rightarrow 0$ limit, in this non-truncated 5D framework, represents effectively a localization of the Higgs scalar on the brane. In contrast, for the regularization I, the physical sense of taking $\epsilon \rightarrow 0$ before having completed the 5D theory (i.e. having taken $N \rightarrow \infty$) is not clear anymore: it is not obvious that it corresponds to the geometric brane-localization along the extra-dimension as it is realized within an hybrid 5D scenario. In other words, this regularization may or may not be equivalent to regularization II. Therefore the experimental tests described above are really necessary to determine whether those two regularizations are experimentally equivalent or not.

The above considerations on the degrees of freedom added by the $Y'_5$, $\tilde{Y}'_5$ parameters are expected to be similar with a warped extra-dimension. Therefore, one can invoke the previous discussion to make the following comments on the past and future literature about the RS scenario (or generally on higher-dimensional theories with a brane-localized Higgs scalar and bulk matter).^15^

As discussed at the beginning of this subsection, regularizations I and II reproduce the present collider data and are thus experimentally equivalent. Hence, the constructions of RS realizations reproducing the fermion masses and mixings performed through regularization I, as for instance in Refs. [63, 65–74], would have been possible as well using regularization II.

Concerning future data, one cannot be sure to predict theoretically all the possible physical values within regularization I (some can be inaccessible as discussed below Eq. (43)) whereas regularization II is clearly exhaustive in its predictions (it includes the parameter space of regularization I which is recovered for $Y'_5=\tilde{Y}'_5= 0$). This is the reason why the RS predictions on KK quark masses, FCNC rates or Higgs productions/decays (involving KK fermion mixings) made e.g. in Refs. [31, 32, 75–77] (4D calculation) [78] (5D calculation)^16^ may not be complete in contrast with those of Refs. [30, 50–52, 54] (5D calculation).

Finally, our recommendations to treat the future experimental data within the RS model are as follows. One should perform regularizations I and II to determine whether in both cases there exist parameters reproducing the whole set of observables (as in Eq. (43)). If regularization I cannot reproduce data then it is excluded, otherwise the two regularizations are experimentally equivalent.^17^ This procedure is important to safely conclude on the validity of these Higgs regularizations and to avoid misleading interpretations. From a practical point of view, the question of the experimental equivalence of these regularizations is also important. Indeed, a systematic calculation of the fermion masses or Yukawa couplings is easier through regularization I than II, both in the 4D (less infinite sums to address cause some mass matrix elements vanish) and 5D (less $\delta (y-\pi R)$ terms in EOM) approaches. Therefore, one could benefit from a regularization equivalence by choosing to use the simpler regularization I.

### The cut-off procedure

Generally speaking, the extra-dimensional backgrounds lead to non-renormalizable theories which are valid only up to a certain energy scale where starts the non-perturbative regime. For instance, in the RS model with bulk matter this scale is driven by the perturbativity of the top Yukawa coupling and is around 2–$3 M_\mathrm{KK}$ ($M_\mathrm{KK}\equiv$ first KK photon mass) (see e.g. Ref. [63]) so that a $\Lambda$ cut-off satisfying, $\Lambda \lesssim 2$–$3 M_\mathrm{KK}$, should be applied. $\Lambda$ indicates the typical energy scale of the UV completion of the theory.

Based on the previous results and discussions, we are going to clarify here the correct and generic way to apply the $\Lambda$ cut-off on scenarios with a Higgs scalar stuck at a brane. Without loss of generality, one should follow this two-step procedure,

1. calculate the bulk fermion mass spectrum and Yukawa couplings including infinite KK tower contributions, as done automatically when manipulating 5D fields or considering infinite mass matrices (with $N \rightarrow \infty$ after/before $\epsilon \rightarrow 0$ accordingly to regularization I/II) in the 4D approach;
2. consider only the obtained mass eigenstates of the towers (masses and couplings derived at step (1)) which are lighter than the $\Lambda$ cut-off, in the computation of physical observables and tree/loop-level amplitudes – with notations of Sect. 4.1, it means that $N_\mathrm{KK}$ must be finite.^18^

The reason for this rigorous order is that one should *first* build formally a consistent and pure 5D theory ($N \rightarrow \infty$) with full KK fermion mixings, *before* truncating this theory at the frontier of its validity domain indicated by $\Lambda$ to get the physical effective low-energy model.

Notice that adopting the inverse order, i.e. (2)$\rightarrow$(1), within regularization II, that is, first, applying the $\Lambda$ cut-off and second, calculating the fermion mass eigenvalues with a finite mass matrix (as the cut-off would prevent from taking $N \rightarrow \infty$) – ending with $\epsilon \rightarrow 0$ – would lead to incomplete eigen-mass expressions (even for the lightest modes) without the $Y'_5$ term (cf. Table 1). Indeed, the non-vanishing contributions from the mass matrix elements involving $Y'_5$ originate non-trivially from the fact that the limit $N \rightarrow \infty$ has been taken (see beginning of Sect. 4.1).

This cut-off procedure is analogous in supersymmetric RS extensions [79] where, at the first step, the 4D effective Lagrangian must be written including infinite KK tower effects: one can then regularize tree-level $\delta (0)$-inconsistencies, arising in the bulk sfermion couplings to two brane-Higgs bosons (from Yukawa and D-terms),^19^ through cancellations with contributions from exchanges of infinite KK towers – treated via the completeness relation. In a second step, one can apply the $\Lambda$ cut-off on tower eigenstates entering the computation e.g. of quantum corrections to the Higgs mass, based on the obtained couplings [79]. This procedure, which has been shown to be the correct one in supersymmetric RS frameworks [79], confirms that one should first elaborate a consistent and thus complete 5D theory (with infinite KK towers) *before* truncating it at the physical cut-off for calculating amplitudes – as justified in previous paragraph.

### Discussion for the square Higgs profile

Let us finally discuss the regularization introduced in Sect. 2.4, which consists in smoothing the Higgs delta peak by a square function. In that case, depending on whether the $(--)$ BC at $\pi R$ is applied before or after solving the EOM system (20a)–(20d) with a square Higgs profile, the mass spectrum is given by Eq. (19) (regularization I) or Eq. (23) (regularization II). In the regularization I, there are no $\Theta$-terms for $(--)$-profiles in Eqs. (20b)–(20c). All this is summarized in Table 2 below, similarly to 5D part of previous Table 1 for the shifted Higgs regularization – except that here $Y_5=Y'_5$ is assumed (case II) for simplicity in the calculation.

**Table 2:**
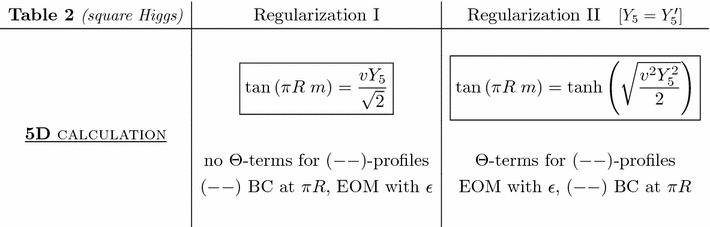
Quark mass spectrum for a square Higgs profile

The Higgs regularizations via a square profile and a shifted delta peak are experimentally equivalent [52, 54, 55] for the same reasons as those presented in detail at the beginning of Sect. 4.3 where regularizations I and II were compared. Note that in the case of regularization I, these two profile regularizations are even formally equivalent as shown by the identical mass spectrum exhibited in Tables 1 and 2. Hence, the above discussion of the equivalence of regularizations I and II (Sect. 4.3) holds also for the square Higgs profile. In particular, the considerations on the counting of degrees of freedom are the same: there are once more additional parameters ($Y'_5$) in regularization II (even if those do not appear explicitly in Table 2 due to the $Y_5=Y'_5$ hypothesis). Finally, the discussion of the cut-off in Sect. 4.4 remains also valid with a square Higgs profile.

### Higher order operators with derivatives

So far in our discussion, we have ignored the possibility that the UV completion of the considered model could induce some higher order operators in the low-energy effective description. In this section, we consider a scenario where higher order operators of the kind

44 $$\begin{aligned}&\delta (y-\pi R) Y_\mathrm{HO} \frac{\partial _y \overline{Q}_R H \partial _y D_L}{\Lambda ^2} \nonumber \\&\quad \Leftrightarrow \delta \left( y-\left[ \pi R-\frac{1}{\Lambda } \right] \right) Y_\mathrm{HO} \bar{Q}_R H \ D_L, \end{aligned}$$

would be present. The motivation is that these specific operators are allowed by the symmetries of the 5D action (1) and are relevant for the present discussion of the regularizations. The term (44) would add up to the 5D action of Eq. (1). Here $\Lambda$ represents the cut-off energy scale, while the $Y_\mathrm{HO}$ coupling constant has the dimension and order of magnitude of *R*. The rewriting as a shifted Yukawa coupling was discussed at the end of Ref. [54] or in Ref. [61].

#### 5D approach

We will show that in the scenario with an operator, like in Eq. (44), regularizations I and II would become analytically equivalent. For that purpose, we will study the effects of such an operator on the fermion mass spectrum, going first through the 5D approach of the Higgs shift procedure. Let us first rewrite the EOM in the presence of such an operator,

45a $$\begin{aligned}&-\ m~q_L - q'_R + \delta (y-\pi R) \frac{v Y_5}{\sqrt{2}}~d_R ~=~ 0, \end{aligned}$$

45b $$\begin{aligned}&-\ m~q_R + q'_L\nonumber \\&\quad +\bigg \{ \delta (y-\pi R) \frac{v Y^\prime _5}{\sqrt{2}} \!+\! \delta \left( y-\left[ \pi R-\frac{1}{\Lambda } \right] \right) \frac{v Y_\mathrm{HO}}{\sqrt{2}} \bigg \}~d_L \!=\! 0, \end{aligned}$$

45c $$\begin{aligned}&-\ m~d_L - d'_R\nonumber \\&\quad +\bigg \{ \delta (y-\pi R) \frac{v Y^\prime _5}{\sqrt{2}} \!+\! \delta \left( y-\left[ \pi R-\frac{1}{\Lambda } \right] \right) \frac{v Y_\mathrm{HO}}{\sqrt{2}} \bigg \}~q_R \!=\! 0, \end{aligned}$$

45d $$\begin{aligned}&-\ m~d_R + d'_L + \delta (y-\pi R) \frac{v Y_5}{\sqrt{2}}~q_L ~=~ 0. \end{aligned}$$

Notice the two additional $Y_\mathrm{HO}$ terms with respect to Eqs. (6a)–(6d).

In the case of regularization I – following the steps described in Sect. 2.3 – the $(--)$ BC assigned to the $d_L$, $q_R$ wave functions have first the effect of eliminating the $\delta (y-\pi R)$ terms in Eqs. (45b)–(45c). One is thus left with,

46a $$\begin{aligned}&-\ m~q_L - q'_R + \delta (y-(\pi -\epsilon ) R) \ \frac{v Y_5}{\sqrt{2}}~d_R = 0, \end{aligned}$$

46b $$\begin{aligned}&-\ m~q_R + q'_L +\delta \left( y-\left[ \pi R-\frac{1}{\Lambda } \right] \right) \frac{v Y^I_\mathrm{HO}}{\sqrt{2}} ~d_L = 0, \end{aligned}$$

46c $$\begin{aligned}&-\ m~d_L - d'_R +\delta \left( y-\left[ \pi R-\frac{1}{\Lambda } \right] \right) \frac{v Y^I_\mathrm{HO}}{\sqrt{2}} ~q_R = 0, \end{aligned}$$

46d $$\begin{aligned}&-\ m~d_R + d'_L + \delta (y-(\pi -\epsilon ) R) \ \frac{v Y_5}{\sqrt{2}}~q_L = 0, \end{aligned}$$

after introducing the regularizing $\epsilon$-shift for the brane-Higgs field. At this level, one can view the inverse cut-off $1/\Lambda$ as the Higgs spatial shift $\epsilon R$, since the limit $\epsilon \rightarrow 0$ imposed by the regularization will then induce the limit $\Lambda \rightarrow \infty$, which must be taken as well – given the step **(1)** described in Sect. 4.4 (infinite KK tower, $N \rightarrow \infty$, to get a pure 5D theory). One thus ends up with the system of Eqs. (8a)–(8d), with $Y^I_\mathrm{HO}$ instead of $Y'_5$. Hence, as in Sect. 2.3, combining the conditions at $(\pi -\epsilon ) R$ (coming from the integrations of the EOM), the wave function expressions and the BC, $\hat{q}_R(\pi R) = \hat{d}_L(\pi R) =0$, leads to the fermion mass spectrum of Eq. (16), with $Y^I_\mathrm{HO}$ instead of $Y'_5$,

47 $$\begin{aligned} \tan \left( \pi R\; m\right) = \frac{\sqrt{2}(1+c)^2 vY_5}{2(1+c)^2+cv^2Y_5Y^I_\mathrm{HO}}. \end{aligned}$$

In the case of regularization II, starting by shifting the brane-Higgs peak in Eqs. (45b)–(45c), one first obtains the EOM,

48a $$\begin{aligned}&-\ m~q_L - q'_R + \delta (y-(\pi -\epsilon ) R) \ \frac{v Y_5}{\sqrt{2}}~d_R ~=~ 0, \end{aligned}$$

48b $$\begin{aligned}&-\ m~q_R + q'_L\nonumber \\&\quad +\bigg \{ \delta (y-(\pi \!-\!\epsilon ) R) \ \frac{v Y^\prime _5}{\sqrt{2}} \!+\! \delta \left( y\!-\!\left[ \pi R\!-\!\frac{1}{\Lambda } \right] \right) \frac{v Y^{II}_\mathrm{HO}}{\sqrt{2}} \bigg \}\nonumber \\&\quad \qquad d_L = 0, \end{aligned}$$

48c $$\begin{aligned}&-\ m~d_L - d'_R\nonumber \\&\quad +\bigg \{ \delta (y-(\pi -\epsilon ) R) \ \frac{v Y^\prime _5}{\sqrt{2}} + \delta \left( y-\left[ \pi R-\frac{1}{\Lambda } \right] \right) \frac{v Y^{II}_\mathrm{HO}}{\sqrt{2}} \bigg \}\nonumber \\&\qquad \quad ~q_R = 0, \end{aligned}$$

48d $$\begin{aligned}&-\ m~d_R + d'_L + \delta (y-(\pi -\epsilon ) R) \ \frac{v Y_5}{\sqrt{2}}~q_L = 0. \end{aligned}$$

Once again, taking the inverse cut-off $1/\Lambda$ as the Higgs shift $\epsilon R$, one ends up with the system of Eqs. (8a)–(8d), with $Y'_5+Y^{II}_\mathrm{HO}$ instead of $Y'_5$. Therefore, as in Sect. 2.3, combining the conditions at $(\pi -\epsilon ) R$, the wave function expressions and the BC, $\hat{q}_R(\pi R) = \hat{d}_L(\pi R) =0$ (Eq. (15)), we find the fermion mass spectrum of Eq. (16), with $Y'_5+Y^{II}_\mathrm{HO}$ instead of $Y'_5$,

49 $$\begin{aligned} \tan \left( \pi R\; m\right) = \frac{\sqrt{2}(1+c)^2 vY_5}{2(1+c)^2+cv^2Y_5 [Y'_5+Y^{II}_\mathrm{HO}]}. \end{aligned}$$

Therefore, the mass spectra from Eq. (47) and Eq. (49) are equal after the parameter redefinition, $Y^{I}_\mathrm{HO} \equiv Y'_5+Y^{II}_\mathrm{HO}$. This means that regularizations I and II are identical in the presence of the higher order operator (44).

#### 4D approach

Similarly, in the 4D approach, the mass matrix element $\beta _{ji}$ of Eq. 31 becomes

50 $$\begin{aligned} \beta _{ji}= & {} \frac{vY^\prime _5}{\sqrt{2}}\, q_R^i ((\pi -\epsilon )R) \times d_L^j((\pi -\epsilon )R)\nonumber \\&+\frac{vY_\mathrm{HO}}{\sqrt{2}}\, q_R^i \left( \pi R-\frac{1}{\Lambda } \right) \times d_L^j \left( \pi R-\frac{1}{\Lambda } \right) . \end{aligned}$$

In regularization I, first applying the regularization limit $\epsilon \rightarrow 0$ would only suppress the $Y^\prime _5$ term of Eq. 50 (since $q^n_R(\pi R) = d^n_L(\pi R)=0$) but not the whole matrix element $\beta _{ji}$. Then following the same calculations in terms of $\beta _{ji}$ as in Sect. 3.2, one writes the matrix CE and works out its infinite KK sums ($N\rightarrow \infty$) leading to the same spectrum as obtained in Eq. 38, except that $Y^\prime _5$ should be replaced there by $Y^{I}_\mathrm{HO}$,

51 $$\begin{aligned} \tan ^2\left( \pi R\sqrt{\vert m \vert ^2}\right)= & {} \left( \frac{4\sqrt{2} vY_5}{8+v^2Y_5Y^{I}_\mathrm{HO}}\right) ^2. \end{aligned}$$

This is guaranteed by the fact that $\beta _{ji} \rightarrow 0$ as well in the limiting case $\Lambda \rightarrow \infty$ (step (1), $N\rightarrow \infty$, of Sect. 4.4).

Within regularization II, one first writes the CE, calculating its KK summations up to $N\rightarrow \infty$. At this level, the $\beta _{ji}$ element of Eq. (50) can be factorized with respect to $Y'_5+Y^{II}_\mathrm{HO}$, given that the Higgs shift $\epsilon R$ is non-vanishing yet and can be taken as the inverse cut-off $1/\Lambda$ (same arguments as in the 5D case). Then imposing the limit $\epsilon \rightarrow 0$, one finds the same spectrum as in Eq. (38), replacing $Y'_5$ by $Y'_5+Y^{II}_\mathrm{HO}$,

52 $$\begin{aligned} \tan ^2\left( \pi R\sqrt{\vert m \vert ^2}\right)= & {} \left( \frac{4\sqrt{2} vY_5}{8+v^2Y_5[Y'_5+Y^{II}_\mathrm{HO}]}\right) ^2. \end{aligned}$$

Once again, the mass spectrum from Eqs. (51) and (52) are equal after the coupling redefinition, $Y^{I}_\mathrm{HO} \equiv Y'_5+Y^{II}_\mathrm{HO}$. We thus recover, using the 4D calculations, the identity of regularizations I and II in the presence of the derivative operator (44).

The conclusion of Sects. 4.6.1 and 4.6.2 reads as follows. In the presence of the higher order operator (44), the non-commutativity disappears and regularizations I and II give rise to the same analytical expression for the fermion mass spectrum. Obviously it is this unique mass spectrum which must be used for phenomenological studies. Notice that this spectrum is identical to the spectrum obtained within regularization II in the absence of higher order operators (see for instance Table 1).

## Summary and conclusions

In the framework of a simple higher-dimensional model with bulk matter and a brane-localized Higgs boson,^20^ we have first pointed out a certain non-commutativity in the order of the 4D calculation for the fermion mass spectrum: applying first the limit $\epsilon \rightarrow 0$ and then $N \rightarrow \infty$ (so-called regularization I) leads to a different analytical expression from the inverse ordering (regularization II). The interpretation of this difference raises obviously a physical question: which order is the correct one?

Then the exact matching between the 4D and 5D calculations of the mass spectrum, which is expected, has been established analytically – for the first time and in both regularizations (I/II). This matching allows a deeper understanding of the regularizations of brane-Higgs models; in particular, it turns out that regularizations I and II differ in their brane-Higgs sensitivity to the tower of bulk $(--)$ profiles for the fermions. Besides, the obtained 4D/5D matching represents another confirmation that the usually applied 5D mixed formalism (i.e. the mixed KK decomposition of Eqs. (3a)–(3d)) is a correct way of including the whole KK mixing effect.

We have further worked out the interpretation of the existence of two types of Higgs peak regularization, which answers the question raised above about the new non-commutativity. The conclusion is that with the present experimental setup, regularizations I and II are experimentally equivalent. Nevertheless, with future constraints from high-energy collider results, it could happen that only regularization I is ruled out – as regularization II involves more free parameters (like $Y'_5$, $\tilde{Y}'_5$). Therefore, there is anyway no regularization-dependence of the model since either regularizations I and II are experimentally equivalent or one of the two is simply excluded.

Our analysis has lead us to clarify the cut-off procedure in models with a brane Higgs: one must *first* build a consistent 5D theory – i.e. calculate eigen-masses and Yukawa couplings accordingly to regularization I or II – with full KK fermion effects ($N \rightarrow \infty$), *before* restricting this theory (finite $N_\mathrm{KK}$) to its validity domain delimited by the $\Lambda$ UV cut-off for computing physical amplitudes. This is analogous to the cut-off process in supersymmetric extensions of the RS model [79].

We mention that even if the Higgs peak regularization used throughout the paper was shifting the delta peak, regularizing the Higgs profile by a smooth square function is experimentally equivalent and has been performed as well. In particular, this square profile treatment has allowed one to confirm our statements on the comparison between regularizations I and II.

Besides, an important complementary result has been found: it has been shown, in the case of the regularization through a Higgs shift, that the non-commutativity disappears in the presence of higher order derivative operators localized on the Higgs brane. In other terms, regularizations I and II have been found to give mass spectra identical to each other – and analytically equivalent to the spectrum from regularization II without higher order operators. Those results hold within both the 4D and the 5D approaches.

## Appendix A: The boundary conditions with boundary terms

In this appendix, we derive BC for the fermions obeying the action of Eq. (1) which contains boundary interactions. Expanding this action, to isolate the terms involving derivatives along the fifth dimension, and integrating by part the second term over the four usual dimensions, one gets,

A.1 $$\begin{aligned} S_{\text {fermion}}= & {} \int \text {d}^4x~\text {d}y~\bigg [ i \bigg ( \bar{Q}\gamma ^\mu \partial _\mu Q ~+ \frac{1}{2}~(\bar{Q}\gamma ^5 \partial _5 Q\nonumber \\&- \partial _5 \bar{Q} \gamma ^5 Q ) + \{ Q \leftrightarrow D \} \bigg )\nonumber \\&+ \delta (y-\pi R) \ (Y_5\ \bar{Q}_LHD_R \nonumber \\&+ Y^\prime _5\ \bar{Q}_RHD_L ~+ \text {H.c.})\bigg ]. \end{aligned}$$

Now integrating by part the third term of this action over the extra dimension and considering the EW symmetry breaking that will affect the mass spectrum, one obtains

A.2 $$\begin{aligned} S_\mathrm{fermion}= & {} \int \mathrm{d}^4x~\mathrm{d}y~\bigg [~ i \bigg ( \bar{Q} \gamma ^\mu \partial _\mu Q + \bar{Q} \gamma ^5 \partial _5 Q \nonumber \\&- \frac{1}{2} ~ \partial _5 (\bar{Q} \gamma ^5 Q) + \{ Q \leftrightarrow D \} \bigg ) \nonumber \\&+ (X_5 \ \bar{Q}_LD_R + X'_5 \ \bar{Q}_RD_L ~+ \text {H.c.}) \bigg ], \end{aligned}$$

with the compact notation, $X_5^{(\prime )} = Y_5^{(\prime )} \frac{v}{\sqrt{2}}\, \delta (y-\pi R)$. Expressing the action in terms of the two-component spinors defined in Eq. (2), Eq. (A.2) becomes

A.3 $$\begin{aligned} S_\mathrm{fermion}= & {} \int \mathrm{d}^4x~\mathrm{d}y~\bigg [\bigg ( -i ~\bar{Q}_R \sigma ^\mu \partial _\mu Q_R ~-i~\bar{Q}_L \bar{\sigma }^\mu \partial _\mu Q_L\nonumber \\&+ \bar{Q}_R \partial _5 Q_L - \bar{Q}_L \partial _5 Q_R \nonumber \\&+ \frac{1}{2} ~ \partial _5 (\bar{Q}_L Q_R-\bar{Q}_R Q_L) + \{ Q \leftrightarrow D \} \bigg )\nonumber \\&+ (X_5 \ \bar{Q}_LD_R + X'_5 \ \bar{Q}_RD_L ~+ \text {H.c.}) \bigg ]. \end{aligned}$$

The variation of the action with respect to the $\bar{Q}_{L,R}$ fields reads

A.4 $$\begin{aligned} \left\{ \begin{array}{l} \delta _{\bar{Q}_L} S_{\text {fermion}}=\int \text {d}^4x~\text {d}y~{[}~ \delta \bar{Q}_L ~ ( -i ~\bar{\sigma }^\mu \partial _\mu Q_L - \partial _5 Q_R + X_5 \ D_R ) ~+ \frac{1}{2} ~ \partial _5 (\delta \bar{Q}_L Q_R) ~ {]} \\ \delta _{\bar{Q}_R} S_{\text {fermion}}=\int \text {d}^4x~\text {d}y~{[}~ \delta \bar{Q}_R ~ ( -i ~\sigma ^\mu \partial _\mu Q_R + \partial _5 Q_L + X'_5 \ D_L ) ~- \frac{1}{2} ~ \partial _5 (\delta \bar{Q}_R Q_L) ~ {]} \end{array} \right. \end{aligned}$$

Analogous equations hold for the $\bar{D}_{L,R}$ spinors (obtained from Eq. (A.4) by the replacements $Q \leftrightarrow D$ and $X_5 \leftrightarrow X_5^{\prime }$). The vanishing for any $\delta \bar{Q}_L$ of the action variation in the first line of Eq. (A.4) is realized, as in the absence of Yukawa interactions, via the separate vanishing of two distinct parts,

A.5 $$\begin{aligned}&-i ~\bar{\sigma }^\mu \partial _\mu Q_L - \partial _5 Q_R + X_5 \ D_R = 0, \end{aligned}$$

A.6 $$\begin{aligned}&\int \text {d}y ~ \partial _5 (\delta \bar{Q}_L Q_R) = [ \delta \bar{Q}_L Q_R ]^{y=\pi R}_{y=0} =0, \end{aligned}$$

the first equation leading to the EOM while the second one defines the BC. By inserting the KK decompositions (3a)–(3d) into Eq. (A.5), and the three similar equations obtained from variations with respect to the 5D fields, $\bar{Q}_R$, $\bar{D}_{L,R}$, one obtains the EOM (6a)–(6d) for the profiles – if one uses the 4D Dirac equations (4)–(5). The condition (A.6) should be satisfied for any $\delta \bar{Q}_L$ so that it can be written, together with all the similar conditions for the other 5D fields,

A.7 $$\begin{aligned}&\delta \bar{Q}_L Q_R \big \vert _{\pi R} = \delta \bar{Q}_L Q_R \big \vert _{0} = \delta \bar{Q}_R Q_L \big \vert _{\pi R} = \delta \bar{Q}_R Q_L \big \vert _{0} = 0, \nonumber \\&\delta \bar{D}_L D_R \big \vert _{\pi R} \!=\! \delta \bar{D}_L D_R \big \vert _{0} \!=\! \delta \bar{D}_R D_L \big \vert _{\pi R} \!=\! \delta \bar{D}_R D_L \big \vert _{0} = 0. \nonumber \\ \end{aligned}$$

The condition, $d_L(0)=q_R(0)=0$, is compatible with Eq. (A.7) – involving the 5D fields (3a)–(3d) – and once inserted into the EOM (6b)–(6c), taken at $y=0$, leads to the additional condition, $d'_R(0)=q'_L(0)=0$ (as the EOM boundary terms are not at $y=0$). Similarly, the condition, $d_L(\pi R)=q_R(\pi R)=0$, respects Eq. (A.7) and after injection in the EOM (6b)–(6c) at $y=\pi R$ gives rise to, $d'_R(\pi R)=q'_L(\pi R)=0$ (here the EOM Yukawa terms are removed by the condition, $d_L(\pi R)=q_R(\pi R)=0$). In summary, the $(--)$ and $(++)$ BC for $d_L,q_R$ and $d_R,q_L$, respectively, fulfill the conditions (A.7)^21^ together with the EOM at the boundaries and thus constitute a satisfactory BC.

We notice that, in order to cancel the action variation, grouping together the terms of the first line of Eq. (A.4) in the following way:

A.8 $$\begin{aligned}&-i ~\bar{\sigma }^\mu \partial _\mu Q_L - \partial _5 Q_R = 0, \nonumber \\&\qquad \times \int \text {d}y ~ [ X_5 \ \delta \bar{Q}_L D_R + \frac{1}{2} ~ \partial _5 (\delta \bar{Q}_L Q_R)]\end{aligned}$$

A.9 $$\begin{aligned}&\quad =\frac{vY_5}{\sqrt{2}}\, \delta \bar{Q}_L D_R\big \vert _{\pi R} + \frac{1}{2} ~ [ \delta \bar{Q}_L Q_R ]^{y=\pi R}_{y=0} =0, \end{aligned}$$

is different from Eqs. (A.5)–(A.6): here, all the boundary terms appear in the same equality (A.9). Equation (A.8), and similar equations for the other fields, would lead to the EOM of Eqs. (4)–(5) and (6a)–(6d) without Yukawa interactions. Those interaction terms would now be included in the Eq. (A.9) which is at the origin of the fermion BC. We see clearly that when one would then try to regularize the Higgs peak, through a shift or a smearing, it would not be possible anymore to write the Yukawa interaction as a boundary term in Eq. (A.9). It means that it is more tricky to follow a regularization procedure when the Yukawa interaction is included in the BC equation (whichever regularization, I or II, is invoked as the $Y_5$ coupling has to be regularized in both). This is the reason why the brane-localized Yukawa interactions are usually (see for instance Ref. [52, 54]) taken into account instead in the EOM, like in our Eq. (A.5). There a shift or a smearing of the Dirac peak contained in $X_5$ does not make the Yukawa coupling disappear from the EOM, since the latter is defined over the whole interval.

## Appendix B: The generic characteristic equation

From the infinite quark mass matrix, [*M*], defined in Eq. (27), we first get the symmetric matrix, $[M^\dag M]$, which can be written without loss of generality as

$$\begin{aligned} \left( \begin{array}{c@{\quad }c@{\quad }c@{\quad }c@{\quad }c@{\quad }c} M_{q_0}^2 +\sum _{n} \beta _{n0}^2 &{}\alpha _{00}M_{q0}+\beta _{00}M_{d0} &{} \sum _{n}\beta _{n0}\beta _{n1} &{} \alpha _{01}M_{q0}+\beta _{10}M_{d1}&{} \sum _{n}\beta _{n0}\beta _{n2} &{} \cdots \\ \vdots &{} M_{d_0}^2 +\sum _{n} \alpha _{n0}^2&{} \alpha _{10}M_{q1}+\beta _{01}M_{d0} &{} \sum _{n}\alpha _{n0}\alpha _{n1}&{}\alpha _{20}M_{q2}+\beta _{02}M_{d0}&{}\cdots \\ \vdots &{} \vdots &{}M_{q_1}^2 +\sum _{n} \beta _{n1}^2 &{} \alpha _{11}M_{q1}+\beta _{11}M_{d1} &{} \sum _{n}\beta _{n1}\beta _{n2} &{}\cdots \\ \vdots &{} \vdots &{} \vdots &{} M_{d_1}^2 +\sum _{n} \alpha _{n1}^2 &{} \alpha _{21}M_{q2}+\beta _{12}M_{d1}&{}\cdots \\ \vdots &{} \vdots &{} \vdots &{} \vdots &{} M_{q_2}^2 +\sum _{n} \beta _{n2}^2 &{} \cdots \\ \vdots &{} \vdots &{} \vdots &{} \vdots &{} \vdots &{} \ddots \end{array} \right) \end{aligned}$$

where the discrete sums are taken from $n=0$ to infinity.

For the CE of this infinite matrix, $[M^\dag M]$, we find the following analytical expression:

B.1 $$\begin{aligned} 1+ \mathcal P_0 + \mathcal P_1=0 \end{aligned}$$

where $\mathcal P_0$ and $\mathcal P_1$ are both series of infinite number of terms. In the limit where all the KK mass (*M*) terms go to zero, $\mathcal P_1$ vanishes but $\mathcal P_0$ does not. In order to describe the parts, $\mathcal P_0$ and $\mathcal P_1$, we have to first define the following structures depending on the $\alpha _{qd}$ (defined in Eq. (28)):

B.2 $$\begin{aligned}&A_n\left( q_1,\ldots , q_n; d_1,\ldots , d_n\right) \nonumber \\&\quad \equiv \sum _{r_1,\ldots ,r_n (\in \{d_1,\ldots , d_n\})} \varepsilon ^{i(r_1)\ldots i(r_n)} \ \varepsilon _{i(d_1)\ldots i(d_n)} \ \alpha _{q_1r_1}\ldots \alpha _{q_nr_n},\nonumber \\ \end{aligned}$$

such that $\varepsilon ^{abc\ldots }$ is the anti-symmetric tensor, with for instance the index $i(r_3) = i(d_2) \hat{=} 2$, and the three first structures can be written explicitly as (notice the specific definition for $A_1\left( q_1; d_1\right)$),

$$\begin{aligned}&A_1\left( q_1; d_1\right) = \alpha _{q_1d_1},\\&A_2\left( q_1,q_2; d_1,d_2\right) = \alpha _{q_1d_1}\alpha _{q_2d_2} - \alpha _{q_1d_2}\alpha _{q_2d_1},\\&A_3\left( q_1,q_2,q_3; d_1,d_2,d_3\right) = \alpha _{q_1d_1}\alpha _{q_2d_2}\alpha _{q_3d_3}\\&\quad + \alpha _{q_1d_2}\alpha _{q_2d_3}\alpha _{q_3d_1} + \alpha _{q_1d_3}\alpha _{q_2d_1}\alpha _{q_3d_2}\\&\quad - \alpha _{q_1d_1}\alpha _{q_2d_3}\alpha _{q_3d_2} -\alpha _{q_1d_3}\alpha _{q_2d_2}\alpha _{q_3d_1} -\alpha _{q_1d_2}\alpha _{q_2d_1}\alpha _{q_3d_3}. \end{aligned}$$

Note that because of the anti-symmetric nature of these structures, in a factorizable case where $\alpha _{qd}=f_q \times f_d$, one has simply

B.3 $$\begin{aligned} A_{n \ge 2} = 0. \end{aligned}$$

Analogous structures can be introduced for the $\beta _{dq}$ (defined in Eq. (29)):

B.4 $$\begin{aligned}&B_n\left( d_1,\ldots , d_n; q_1,\ldots , q_n\right) \nonumber \\&\quad \equiv \sum _{r_1,\ldots ,r_n ( \in \{d_1,\ldots , d_n\})} \varepsilon ^{i(r_1)\ldots i(r_n)} \ \varepsilon _{i(d_1)\ldots i(d_n)} \beta _{r_1q_1}\ldots \beta _{r_nq_n} . \end{aligned}$$

One has similarly, $B_{n \ge 2} = 0$, for factorizable cases with, $\beta _{dq}=f_d \times f_q$. Now, with the conditions

B.5 $$\begin{aligned} q_i \ne q_j \ne Q_k&;&d_i \ne d_j \ne D_k \end{aligned}$$

where $i,j,k = 0,1,2, \ldots$, the first few terms (sufficient to deduce the rest of the infinite series) in the $\mathcal P_0$ and $\mathcal P_1$ parts can be expressed as

$$\begin{aligned} \mathcal P_0= & {} \sum _{q_1;d_1}(-\lambda )\left( \frac{\left( A_1(q_1;d_1)\right) ^2}{(M_{q_1}^2-\lambda )(M_{d_1}^2-\lambda )} +(\alpha \leftrightarrow \beta )\right) \\&+\sum _{q_1<q_3;d_1<d_3}(-\lambda )^2\\&\times \left( \frac{\left( A_2(q_1,q_3;d_1,d_3)\right) ^2}{(M_{q_1}^2-\lambda )(M_{d_1}^2-\lambda )(M_{q_3}^2-\lambda )(M_{d_3}^2-\lambda )} \right. \\&\left. +(\alpha \leftrightarrow \beta )\right) +\sum _{q_1,q_2;d_1,d_2}(-\lambda )^2 \ \frac{\left( A_1(q_1;d_1)\right) ^2}{(M_{q_1}^2-\lambda )(M_{d_1}^2-\lambda )}\\&\times \frac{\left( B_1(d_2;q_2)\right) ^2}{(M_{q_2}^2-\lambda )(M_{d_2}^2-\lambda )}\\&\times \left( 1-\delta _{q_1q_2}\frac{M_{q_2}^2}{\lambda }\right) \left( 1-\delta _{d_1d_2}\frac{M_{d_2}^2}{\lambda }\right) \\&+\sum _{q_1<q_3,q_2;d_1<d_3,d_2}(-\lambda )^3\\&\times \left\{ \frac{\left( A_2(q_1,q_3;d_1,d_3)\right) ^2}{\prod _{i=1,3}(M_{q_i}^2-\lambda )(M_{d_i}^2-\lambda )}\right. \\&\times \frac{\left( B_1(d_2;q_2)\right) ^2}{(M_{q_2}^2-\lambda )(M_{d_2}^2-\lambda )}\\&\times \left( 1-\delta _{q_1q_2}\frac{M_{q_2}^2}{\lambda }\right) \left( 1-\delta _{d_1d_2}\frac{M_{d_2}^2}{\lambda }\right) \\&\times \left. \left( 1-\delta _{q_3q_2}\frac{M_{q_2}^2}{\lambda }\right) \left( 1-\delta _{d_3d_2}\frac{M_{d_2}^2}{\lambda }\right) +(\alpha \leftrightarrow \beta )\right\} \\&+\sum _{q_1<q_3,q2<q_4;d_1<d_3,d_2<d_4}(-\lambda )^4 \\&\times \frac{\left( A_2(q_1,q_3;d_1,d_3)\right) ^2}{\prod _{i=1,3}(M_{q_i}^2-\lambda )(M_{d_i}^2-\lambda )} \end{aligned}$$

B.6 $$\begin{aligned}&\times \frac{\left( B_2(d_2,d_4;q_2,q_4)\right) ^2}{\prod _{j=2,4}(M_{q_j}^2-\lambda )(M_{d_j}^2-\lambda )}\nonumber \\&\times \left( 1-\delta _{q_1q_2}\frac{M_{q_2}^2}{\lambda }\right) \left( 1-\delta _{q_3q_2}\frac{M_{q_2}^2}{\lambda }\right) \nonumber \\&\times \left( 1-\delta _{q_1q_4}\frac{M_{q_4}^2}{\lambda }\right) \left( 1-\delta _{q_3q_4}\frac{M_{q_4}^2}{\lambda }\right) \nonumber \\&\times \left( 1-\delta _{d_1d_2}\frac{M_{d_2}^2}{\lambda }\right) \left( 1-\delta _{d_3d_2}\frac{M_{d_2}^2}{\lambda }\right) \nonumber \\&\times \left( 1-\delta _{d_1d_4}\frac{M_{d_4}^2}{\lambda }\right) \left( 1-\delta _{d_3d_4}\frac{M_{d_4}^2}{\lambda }\right) \nonumber \\&+ \mathcal O(\alpha ^6,\beta ^6) \end{aligned}$$

and

$$\begin{aligned} \mathcal P_1= & {} \sum _{Q_1;D_1} \frac{-2 M_{Q_1}M_{D_1}}{(M_{Q_1}^2-\lambda )(M_{D_1}^2-\lambda )}\\&\times \left[ A_1(Q_1;D_1)\times B_1(D_1;Q_1)\right. \\&+\sum _{q_1;d_1} (-\lambda )\left\{ \frac{A_2(Q_1,q_1;D_1,d_1)A_1(q_1;d_1) }{(M_{q_1}^2-\lambda )(M_{d_1}^2-\lambda )}\right. \\&\times \left. B_1(D_1;Q_1) + (\alpha \leftrightarrow \beta )\right\} \\&+\sum _{q_1q_2;d_1,d_2} (-\lambda )^2\times \frac{A_2(Q_1,q_1;D_1,d_1)A_1(q_1;d_1)}{(M_{q_1}^2-\lambda )(M_{d_1}^2-\lambda )}\\&\times \frac{B_2(D_1,d_2;Q_1,q_2)B_1(d_2;q_2)}{(M_{q_2}^2-\lambda )(M_{d_2}^2-\lambda )} \\&\left. \times \left( 1-\delta _{q_1q_2}\frac{M_{q_2}^2}{\lambda }\right) \left( 1-\delta _{d_1d_2}\frac{M_{d_2}^2}{\lambda }\right) + \mathcal O(\alpha ^5, \beta ^5)\right] \\&+\left\{ \sum _{Q_1<Q_2} \frac{2 M_{Q_1}M_{Q_2}}{(M_{Q_1}^2-\lambda )(M_{Q_2}^2-\lambda )}\right. \\&\times \left[ \sum _{d_1,d_2} (-\lambda )\times \frac{A_1(Q_1;d_1)A_1(Q_2;d_1)}{(M_{d_1}^2-\lambda )}\right. \\&\times \frac{ B_1(d_2;Q_1)B_1(d_2;Q_2)}{(M_{d_2}^2-\lambda )}\left( 1-\delta _{d_1d_2}\frac{M_{d_2}^2}{\lambda }\right) \\&+\sum _{q_1;d_1,d_2,d_3}(-\lambda )^2 \\&\times \left\{ \frac{A_2(Q_1,q_1;d_1,d_3) A_2(Q_2,q_1;d_1,d_3)}{(M_{q_1}^2-\lambda )(M_{d_1}^2-\lambda )}\right. \\&\left. \times \frac{B_1(d_2;Q_1)B_1(d_2;Q_2)}{(M_{d_3}^2-\lambda )}\right. \\&\times \left. \left( 1-\delta _{d_1d_2}\frac{M_{d_2}^2}{\lambda }\right) \left( 1-\delta _{d_3d_2}\frac{M_{d_2}^2}{\lambda }\right) + (\alpha \leftrightarrow \beta )\right\} \\&+\sum _{q_1,q_2;d_1,d_2,d_3,d_4}(-\lambda )^3 \end{aligned}$$

$$\begin{aligned}&\times \frac{A_2(Q_1,q_1;d_1,d_3) A_2(Q_2,q_1;d_1,d_3)}{(M_{q_1}^2-\lambda )(M_{d_1}^2-\lambda )(M_{d_3}^2-\lambda )}\\&\times \frac{B_2(d_2,d_4;Q_1,q_2)B_2(d_2,d_4;Q_2,q_2)}{(M_{q_2}^2-\lambda )(M_{d_2}^2-\lambda )(M_{d_4}^2-\lambda )}\\&\times \left( 1-\delta _{q_1q_2}\frac{M_{q_2}^2}{\lambda }\right) \left( 1-\delta _{d_1d_2}\frac{M_{d_2}^2}{\lambda }\right) \left( 1-\delta _{d_1d_4}\frac{M_{d_4}^2}{\lambda }\right) \\&\left. \times \left( 1-\delta _{d_3d_2}\frac{M_{d_2}^2}{\lambda }\right) \left( 1-\delta _{d_3d_4}\frac{M_{d_4}^2}{\lambda }\right) + \mathcal O(\alpha ^6, \beta ^6)\right] \\&\left. +\left[ (Q_1,Q_2) \rightarrow (D_1,D_2)\right] \right\} \\&+\sum _{Q_1<Q_2;D_1<D_2} 2\left( \prod _{i=1,2}\frac{ M_{Q_i}M_{D_i}}{(M_{Q_i}^2-\lambda )(M_{D_i}^2-\lambda )}\right) \\&\times [ A_2(Q_1,Q_2;D_1,D_2)\times B_2(D_1,D_2;Q_1,Q_2) \\&+ \ A_1(Q_1;D_1)A_1(Q_2;D_2)\times B_1(D_1;Q_1)B_1(D_2;Q_2) \\&+ \ A_1(Q_1;D_2)A_1(Q_2;D_1)\\&\times B_1(D_2;Q_1)B_1(D_1;Q_2) + \mathcal O(\alpha ^4, \beta ^4)]\\&+\left\{ \sum _{Q_1<Q_2<Q_3;D_1} (-2)\left( \prod _{i=1,3}\frac{ M_{Q_i}}{(M_{Q_i}^2-\lambda )}\right) \frac{M_{D_1}}{(M_{D_1}^2-\lambda )}\right. \\&\times \left[ \sum _{d_1,d_2}(-\lambda )\Bigg ( \frac{A_2(Q_1,Q_2;D_1,d_1 )A_1(Q_3;d_1)}{(M_{d_1}^2-\lambda )}\right. \end{aligned}$$

$$\begin{aligned}&\times \frac{B_2(D_1,d_2;Q_1,Q_2)B_1(d_2;Q_3)}{(M_{d_2}^2-\lambda )}\\&+\frac{A_2(Q_1,Q_3;D_1,d_1 )A_1(Q_2;d_1)}{(M_{d_1}^2-\lambda )}\\&\times \frac{B_2(D_1,d_2;Q_1,Q_3)B_1(d_2;Q_3)}{(M_{d_2}^2-\lambda )}\\&+\frac{A_2(Q_2,Q_3;D_1,d_1 )A_1(Q_1;d_1)}{(M_{d_1}^2-\lambda )}\\&\times \frac{B_2(D_1,d_2;Q_2,Q_3)B_1(d_2;Q_1)}{(M_{d_2}^2-\lambda )} \Bigg )\\&\left. \times \left( 1-\delta _{d_1d_2}\frac{M_{d_2}^2}{\lambda }\right) + \mathcal O(\alpha ^5, \beta ^5) \right] \\&\left. +(M_Q\leftrightarrow M_D, \alpha \leftrightarrow \beta )\right\} \\&+\left\{ \sum _{Q_1<Q_2<Q_3<Q_4} 2\left( \prod _{i=1,4}\frac{ M_{Q_i}}{(M_{Q_i}^2-\lambda )}\right) \right. \\&\times \left[ \sum _{d_1,d_2}(-\lambda )^2\Bigg ( \frac{A_2(Q_1,Q_2;d_1,d_3 )A_2(Q_3,Q_4;d_1,d_3)}{(M_{d_1}^2-\lambda )(M_{d_3}^2-\lambda )}\right. \\&\times \frac{B_2(d_2,d_4;Q_1,Q_2)B_2(d_2,d_4;Q_3,Q_4)}{(M_{d_2}^2-\lambda )(M_{d_4}^2-\lambda )}\\&+\frac{A_2(Q_1,Q_3;d_1,d_3 )A_2(Q_2,Q_4;d_1,d_3)}{(M_{d_1}^2-\lambda )(M_{d_3}^2-\lambda )}\\&\times \frac{B_2(d_2,d_4;Q_1,Q_3)B_2(d_2,d_4;Q_2,Q_4)}{(M_{d_2}^2-\lambda )(M_{d_4}^2-\lambda )} \end{aligned}$$

B.7 $$\begin{aligned}&+\frac{A_2(Q_1,Q_4;d_1,d_3 )A_2(Q_2,Q_3;d_1,d_3)}{(M_{d_1}^2-\lambda )(M_{d_3}^2-\lambda )}\nonumber \\&\times \frac{B_2(d_2,d_4;Q_1,Q_4)B_2(d_2,d_4;Q_2,Q_3)}{(M_{d_2}^2-\lambda )(M_{d_4}^2-\lambda )}\Bigg )\nonumber \\&\times \left( 1-\delta _{d_1d_2}\frac{M_{d_2}^2}{\lambda }\right) \left( 1-\delta _{d_1d_4}\frac{M_{d_4}^2}{\lambda }\right) \nonumber \\&\left. \times \left( 1-\delta _{d_3d_2}\frac{M_{d_2}^2}{\lambda }\right) \left( 1-\delta _{d_3d_4}\frac{M_{d_4}^2}{\lambda }\right) + \mathcal O(\alpha ^6, \beta ^6) \right] \nonumber \\&\left. +(M_Q\leftrightarrow M_D, \alpha \leftrightarrow \beta ) \right\} , \end{aligned}$$

where all the discrete sums are taken from zero to infinity and $\lambda$ represents the eigenvalues of the $[M^\dag M]$ matrix.

## Footnotes

1 The third family of heavy SM quarks is generally expected to feel the largest mixings with KK modes, but our formalism is directly extendable to any quark generation as well as to leptons.

2 Although we have kept $\beta _{0j}$, $\beta _{i0}$ in the matrix (27) to make its ($\alpha _{ij} \leftrightarrow \beta _{ji}$) symmetric texture explicit, we note that $\beta _{0j} = \beta _{i0} = 0$ since the zero-modes $q^0_R(y)=d^0_L(y) =0$.

3 Due to cancellations among different terms, Eq. 37 does depend ultimately neither on $\gamma$ nor on $\log (-i\epsilon )$.

4 In a preliminary work [56] on the RS framework, an approximated mass spectrum was obtained in this ordering ($\epsilon \rightarrow 0$, $N \rightarrow \infty$) through an expansion in powers of $v^2/M_\mathrm{KK}^2$.

5 See also Refs. [57–59].

6 It was pointed out [61], based on a 4D calculation of the gluon–gluon fusion amplitude in RS, that some specific higher derivative operators allow one to take into account a UV sensitivity.

7 In the 4D limit $N \rightarrow \infty$, the effect of the infinite KK tower is taken into account which is equivalent to consider rigorously the full 5D fields of Eqs. (24a)–(24d).

8 The precise notion of experimentally equivalent regularizations will be described at the beginning of Sect. 4.3.

9 As the renormalizations of quantum corrections were confronted (with success) to collider data.

10 The expression “experimentally equivalent”, used here and throughout the paper, is motivated by the fact that the two regularizations can give rise to the same value of a measured mass *m*. Now it is clear that, formally speaking, regularization II may generate values of a mass observable in a range different from regularization I (as can be seen by comparing the mass spectra in Table 1), and, the degrees of freedom involved in the mass spectrum are also different (appearance or not of the $Y'_5$ coupling constant); in this sense regularizations I and II are not “physically equivalent”.

11 Basically different masses depend on different $Y_5$-like parameters (i.e. $Y_5$, $\tilde{Y}_5$, $Y_{\ell 5}$,...).

12 Going from this toy model to RS [11], one should add the $AdS_5$ curvature parameter, *k*, but $kR\approx 11$ is fixed by the gauge hierarchy solution. For the RS custodially protected version [62] there can be an additional freedom from the $\tilde{M}$ parameter of explicit bulk custodial symmetry breaking, or even another one via the $g_{Z'}$ coupling [63] in case of no left–right parity [64]. One should also add basically the 5D mass parameters, $c_{u,d,\nu ,l}^{L/R \ i}$ ($i=1,2,3$), in the RS extensions addressing the flavor problem [65–74].

13 For instance with $f(c_q)\ll 1$, $f(-c_d)\ll 1$ and $Y'_5\ll Y_5$.

14 In the 4D approach, this notation is coherent with previous notations if the $D^m_R(x)$ fields result from a mixing with the $Q^m_R(x)$ fields, and the $Q^n_L(x)$ include mixings with $D^n_L(x)$.

15 For constructions of RS scenarios with a brane Higgs as a limit case of bulk-Higgs models, we refer to Refs. [53, 54].

16 Let us also mention Ref. [56] in regularization I, which presents 4D/5D matching considerations via a numerical approach and for a truncated KK fermion tower.

17 The last possible situation with both regularizations unable to reproduce data would mean that either another kind of regularization is necessary (as both previous regularizations would then not be correct) or the RS model itself (in its minimal version with an Higgs boson strictly localized on the brane) is ruled out.

18 Even if a cut-off should be applied on physical observables, it may be instructive to take the limit $N_\mathrm{KK}\rightarrow \infty$ for technical purposes in formal discussions on the calculation itself [30, 50–53].

19 And in self-couplings of the Higgs bosons as well.

20 Our results are expected to be essentially similar (up to warp factors of course) in realistic warped extra-dimension scenarios.
